# Intra-specific variation in sensitivity of *Bombus terrestris* and *Osmia bicornis* to three pesticides

**DOI:** 10.1038/s41598-022-22239-4

**Published:** 2022-10-15

**Authors:** Alberto Linguadoca, Margret Jürison, Sara Hellström, Edward A. Straw, Peter Šima, Reet Karise, Cecilia Costa, Giorgia Serra, Roberto Colombo, Robert J. Paxton, Marika Mänd, Mark J. F. Brown

**Affiliations:** 1 Centre for Ecology, Evolution & Behaviour, Department of Biological Sciences, School for Life Sciences and the Environment, Royal Holloway University of London, Egham, UK; 2 Pesticide Peer Review Unit, European Food Safety Authority (EFSA), via Carlo Magno 1A, 43126 Parma, Italy; 3 Chair of Plant Health, Institute of Agricultural and Environmental Sciences, Estonian University of Life Sciences, Tartu, Estonia; 4 General Zoology, Institute for Biology, Martin Luther University Halle-Wittenberg, Halle (Saale), Germany; 5 Department of R&D, Koppert s.r.o., Nové Zámky, Slovakia; 6 CREA Research Centre for Agriculture and Environment, via di Corticella 133, 40128 Bologna, Italy

**Keywords:** Environmental sciences, Conservation biology

## Abstract

There is growing evidence that pesticides may be among the causes of worldwide bee declines, which has resulted in repeated calls for their increased scrutiny in regulatory assessments. One recurring concern is that the current frameworks may be biased towards assessing risks to the honey bee. This paradigm requires extrapolating toxicity information across bee species. Most research effort has therefore focused on quantifying differences in sensitivity across species. However, our understanding of how responses to pesticides may vary within a species is still very poor. Here we take the first steps towards filling this knowledge gap by comparing acute, lethal hazards in sexes and castes of the eusocial bee *Bombus terrestris* and in sexes of the solitary bee *Osmia bicornis* after oral and contact exposure to the pesticides sulfoxaflor, Amistar (azoxystrobin) and glyphosate. We show that sensitivity towards pesticides varies significantly both within and across species. Bee weight was a meaningful predictor of pesticide susceptibility. However, weight could not fully explain the observed differences, which suggests the existence of unexplored mechanisms regulating pesticide sensitivity across bee sexes and castes. Our data show that intra-specific responses are an overlooked yet important aspect of the risk assessment of pesticides in bees.

## Introduction

There is growing consensus that anthropogenic activities are causing the worldwide decline of wild bees^1–3^. The mechanisms regulating this phenomenon are likely multifactorial and interactive^4,5^. However, there is growing evidence that, among these stressors, the role of pesticides may be pivotal^4–6^. As bees are key pollinators^7^, their loss may affect food system stability^8,9^ and biodiversity^10^, with regionally unsustainable consequences^6^. Increasing awareness of the role of pesticides in this paradigm has sparked growing interest in how their risks are characterised before they enter the market^11^ and how pesticides are used following regional authorisation^12^.

One recurring concern is that environmental risk assessment, because of its reductionist nature, is centred towards testing standard model species^13^. In the case of risk assessment of bee pollinators, honey bees are widely used as a standard model species. In other words, pesticide hazards characterised using this model species are extrapolated to all other bee taxa, which are not routinely tested^11^. The uncertainty resulting from such inference is counterbalanced by using assessment factors^14,15^, which are meant to correct for the inter-specific variation of responses.

Many studies have investigated whether such assessment factors may be sufficiently protective^16–19^ or not^20,21^ of particularly sensitive species. Likewise, efforts have been made to characterise how differences in bee ecology and life history traits across species may influence bee vulnerability to pesticide exposure, encouraging further investigation of pesticide risks across species other than the honey bee^22–25^. In contrast, data investigating intra-specific acute sensitivity to pesticides, i.e., between sexes and, in the case of eusocial species, castes are surprisingly scarce. By inference, it might be assumed that intraspecific variation in sensitivity to pesticides may be largely explained by size differences across castes and sexes, as is often the case for interspecific sensitivity differences^17^. However, neonicotinoid exposure has been shown to trigger differential expression of detoxification^26,27^, immune^28^, and stress response^29^ genes across bee castes and sexes, which suggests factors other than body weight may similarly regulate intraspecific sensitivity.

Here we characterise the acute, lethal hazard of three pesticides upon oral and contact exposure in the eusocial buff-tailed bumble bee (*Bombus terrestris* L.) and the solitary red mason bee (*Osmia bicorni*s L. Syn. *Osmia rufa* L.) following ring-tested study designs. *B. terrestris* and *O. bicornis* were used as candidate species because—unlike honey bees—their sexes and castes have a similar likelihood of exposure in the field^23–25^. Moreover, these species have been proposed as suitable models by recent regulatory risk assessment schemes^14^, and recent standardised acute testing protocols^30–32^.

We used the sulfoximine insecticide sulfoxaflor, the methoxy-acrylate fungicide Amistar (azoxystrobin 250 g/l, Suspension Concentrate, see supplementary methods, S1) and the glycine herbicide glyphosate (as active substance, RoundUp ProActive or RoundUp FL, see supplementary methods, S1) as model substances. Our choice was justified by their widespread use, regulatory status and systemic uptake in plants. Because of these characteristics, the likelihood of bees being exposed in the field was considered similarly plausible across model substances. Additionally, we are not aware of published evidence of the acute toxicity of these substances across castes and sexes of *B. terrestris* and *O. bicornis.*

Using these model substances, we performed a comparative analysis of acute lethal hazards across sexes and castes of *B. terrestris* and *O. bicornis* by analysing sensitivity ratios (SR) between median lethal doses (LD_50_)^33,34^.

As we are not aware of published studies that have systematically quantified pesticide toxicity across sexes and castes of bee species upon different exposure routes, here we address this knowledge gap by testing the following hypotheses: (i) that the bee species *B. terrestris* and *O. bicornis* show different levels of acute pesticide sensitivity; (ii) that the sensitivity across castes and sexes within these two species varies; and (iii) that body size per se does not explain intraspecific variation in sensitivity.

## Results

The positive control dimethoate and the negative control in all dose response analyses were in line with standardised study protocols^30,31^ at timepoints selected for the comparative analysis. Specifically, the positive control caused mortality above 50%, while the negative control caused below 10% mortality for bumble bees and 15% for *O. bicornis*^35–37^.

### *O. bicornis* and *B. terrestris* show differential sensitivity to sulfoxaflor and Amistar

**Figure 1:**
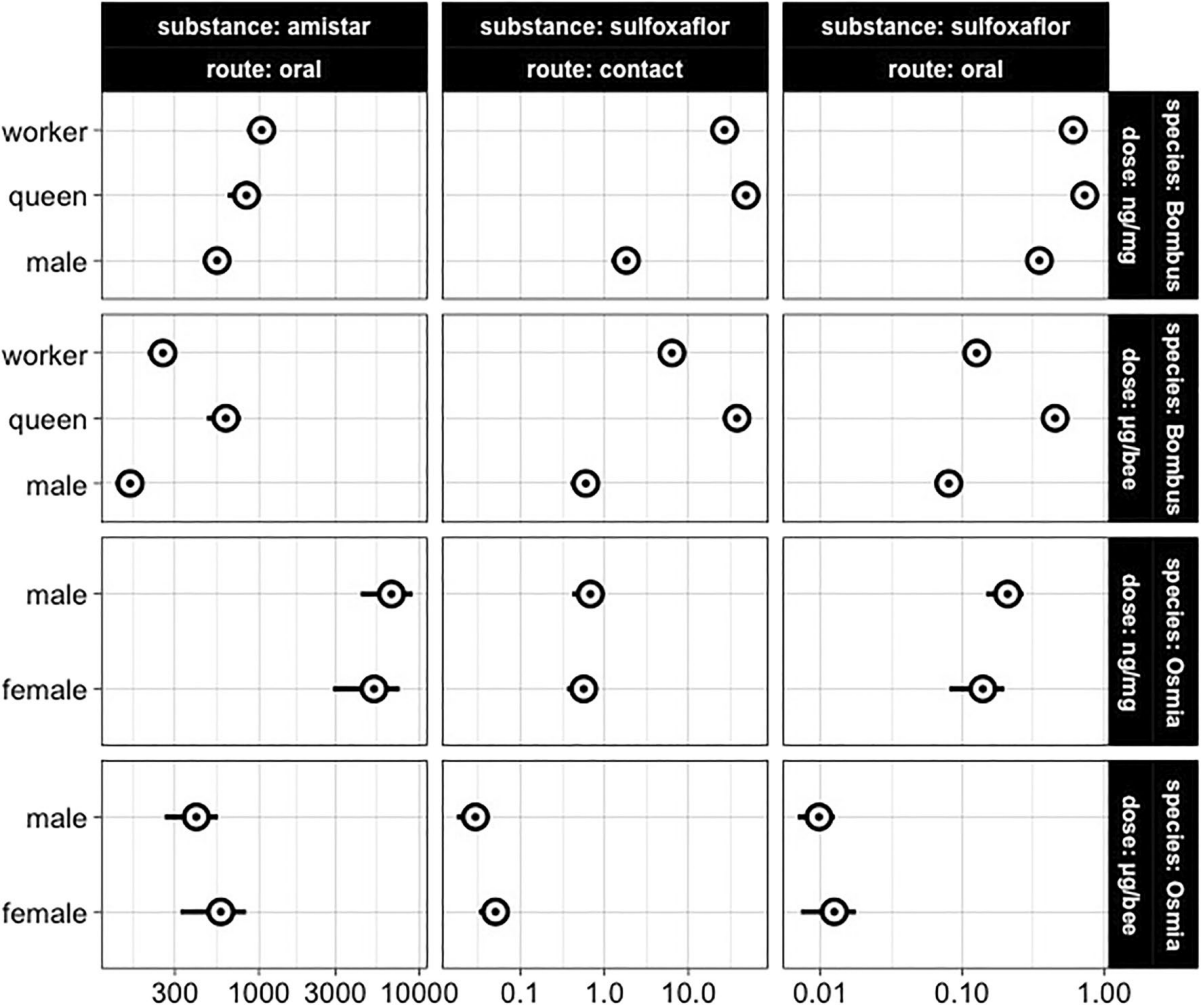
The acute toxicity of Amistar (oral) and sulfoxaflor (oral and contact) across sexes and castes of *B. terrestris* and *O. bicornis.* Dots represent the median lethal doses, while horizontal error bars represent the 95% confidence intervals. The y-axis lists the bee castes and sexes, while the x-axis reports the doses (i.e., non-normalised = µg/bee or normalised = ng/mg body weight) on a logarithmic scale.

*Osmia bicornis* females were more sensitive to sulfoxaflor than bumble bee workers, both before and after doses were normalised by body weight (Tables 1, 2; Fig. 1).

**Table 1:** The acute toxicity of sulfoxaflor and Amistar to *B. terrestris* and *O. bicornis* across castes and sexes.

Sex/caste	Timepoint (h)	µg/bee (CI)	ng/mg (CI)
**Sulfoxaflor LD**_**50**_ ***Bombus*** ***terrestris* ssp. *terrestris* contact**			
Worker	48	6.323 (4.672, 7.975)	26.741 (19.771, 33.71)
Male	72	0.6 (0.399, 0.801)	1.821 (1.214, 2.428)
Queen	96	37.513 (28.775, 46.25)	48.038 (36.867, 59.208)
**Sulfoxaflor LD**_**50**_ ***Bombus*** ***terrestris* ssp. *audax* oral**			
Worker	48	0.126 (0.12, 0.133)	0.606 (0.581, 0.631)
Male	48	0.08 (0.066, 0.095)	0.35 (0.289, 0.411)
Queen	96	0.452 (0.369, 0.535)	0.731 (0.605, 0.857)
**Amistar LD**_**50**_ ***Bombus*** ***terrestris* ssp. *audax* oral**			
Worker	96	252.3 (201.6, 303.0)	1043.2 (837.7, 1248.7)
Male	72	157.6 (128.2, 186.9)	549 (448.913, 649.0)
Queen	96	622.6 (473.7, 771.6)	836.9 (639, 1034.8)
**Sulfoxaflor LD**_**50**_ ***Osmia*** ***bicornis* contact**			
Female	96	0.051 (0.032, 0.069)	0.567 (0.358, 0.775)
Male	96	0.029 (0.018, 0.041)	0.679 (0.413, 0.946)
**Sulfoxaflor LD**_**50**_ ***Osmia*** ***bicornis* oral**			
Female	48	0.013 (0.007, 0.018)	0.140 (0.081, 0.199)
Male	48	0.01 (0.007, 0.013)	0.209 (0.147, 0.271)
**Amistar LD**_**50**_ ***Osmia*** ***bicornis* oral**			
Female	48	578.8 (324.4, 833.2)	5324 (2902.9, 7565.2)
Male	48	407.4 (258.5, 556.4)	6715.8 (4314.4, 9117.3)

LD_50_ values and related 95% confidence intervals were calculated at timepoints corresponding to the steady-state mortality except in the case of *O. bicornis* oral exposure to sulfoxaflor and Amistar (see methods section). All endpoints are expressed as doses, normalised or not by body weight.

**Table 2:** The Sensitivity Ratio (SR) and comparison across castes and sexes of *B. terrestris* and *O. bicornis* upon acute exposure to sulfoxaflor (oral and contact) and Amistar (oral).

Sex/caste	Timepoint (h)	SR µg/bee (CI)	SR ng/g (CI)
**Sulfoxaflor LD**_**50**_ ***Bombus*** ***terrestris* ssp. *terrestris* contact**			
Worker/queen	48/96	0.17 (0.11, 0.23)	0.56 (0.36, 0.75)
Worker/male	48/72	10.54 (6.06, 15.02)	14.68 (8.47, 20.89)
Male/queen	72/96	0.02 (0.01, 0.02)	0.04 (0.02, 0.05)
**Sulfoxaflor LD**_**50**_ ***Bombus*** ***terrestris* ssp. *audax* oral**			
Worker/queen	48/96	0.28 (0.23, 0.33)	0.83 (0.68, 0.98)
Worker/male	48/48	1.57 (1.28, 1.87)	1.73 (1.42, 2.04)
Male/queen	48/96	0.18 (0.13, 0.22)	0.48 (0.36, 0.6)
**Amistar LD**_**50**_ ***Bombus*** ***terrestris* ssp. *audax* oral**			
Worker/queen	96/96	0.41 (0.28, 0.53)	1.25 (0.86, 1.63)
Worker/male	96/72	1.6 (1.16, 2.04)	1.9 (1.39, 2.41)
Male/queen	72/96	0.25 (0.18, 0.33)	0.66 (0.46, 0.85)
**Sulfoxaflor LD**_**50**_ ***Osmia*** ***bicornis* contact**			
Female/male	96/96	1.73 (0.79, 2.67)	0.83 (0.39, 1.28)
**Sulfoxaflor LD**_**50**_ ***Osmia*** ***bicornis* oral**			
Female/male	48/48	1.28 (0.63, 1.94)	0.67 (0.32, 1.01)
**Amistar LD**_**50**_ ***Osmia*** ***bicornis* oral**			
Female/male	48/48	1.42 (0.61, 2.23)	0.78 (0.33, 1.22)
**Sulfoxaflor LD**_**50**_ ***Osmia*** ***bicornis/Bombus*** ***terrestris* ssp. *audax* oral**			
Female/worker	48/48	0.10 (0.06, 0.14)	0.23 (0.13, 0.33)
Male/male	48/48	0.12 (0.08, 0.16)	0.6 (0.39, 0.80)
**Sulfoxaflor LD**_**50**_ ***Osmia*** ***bicornis/Bombus*** ***terrestris* ssp. *terrestris* contact**			
Female/worker	96/48	0.008 (0.004, 0.012)	0.02 (0.01, 0.03)
Male/male	48/72	0.05 (0.02, 0.07)	0.37 (0.18, 0.56)
**Amistar LD**_**50**_ ***Osmia*** ***bicornis/Bombus*** ***terrestris* ssp. *audax* oral**			
Female/worker	48/96	2.29 (1.18, 3.40)	5.02 (2.57, 7.46)
Male/male	48/72	2.59 (1.52, 3.65)	12.23 (7.32, 17.14)

SRs and related 95% confidence intervals were calculated using data reported in Fig. 1. Timepoints correspond to the steady-state mortality (less than 10% increase across 24 h) except in the case of *O. bicornis* oral exposure to sulfoxaflor and Amistar.

Similarly, *O. bicornis* males were more sensitive to sulfoxaflor than *B. terrestris* males both before and after doses were normalised by body weight (Tables 1, 2; Fig. 1).

Conversely, upon oral exposure to Amistar, *O. bicornis* was less sensitive than *B. terrestris* before and after normalisation by body weight (Tables 1, 2; Fig. 1).

### Responses across castes and sexes differ for reasons other than body weight in *B. terrestris*

**Figure 2:**
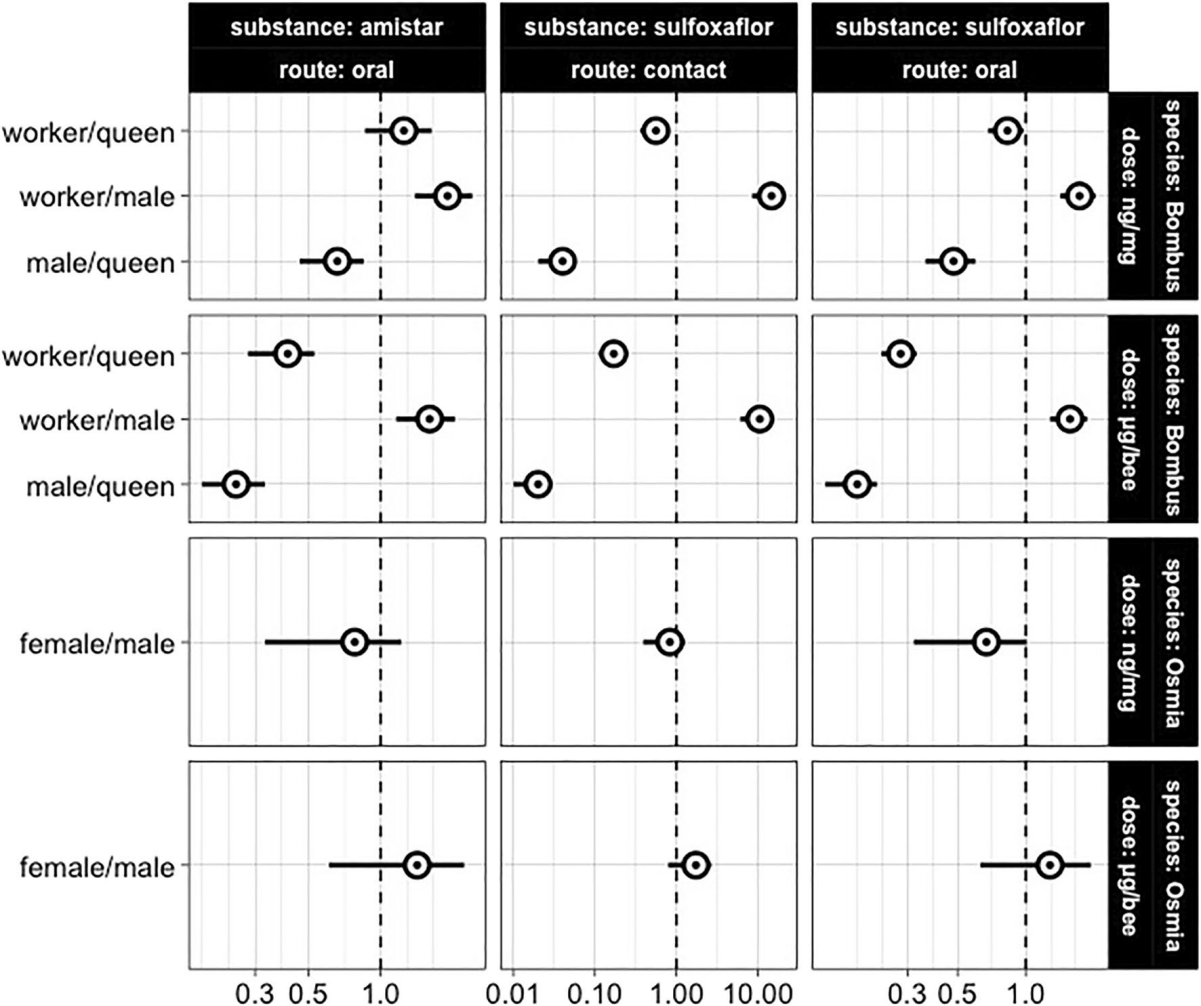
The intra-specific sensitivity across sexes and castes of *B. terrestris* and *O. bicornis.* Dots represent the Sensitivity Ratios (SR), while error bars represent the 95% confidence interval. The y-axis reports the relevant comparisons, while the x-axis reports the SR (dimensionless). The vertical dashed line (SR = 1) represents the case where the two compared LD_50s_ are equal, below which the sensitivity is higher. SRs are considered significant when their confidence bounds do not cross 1.

Bumble bee queens were less sensitive than workers and males upon contact and oral exposure to sulfoxaflor and oral exposure to Amistar, before doses were normalised by bee weight (Table 2; Fig. 2). For sulfoxaflor, such differences were smaller but remained significantly different after doses were normalised by body weight.

For Amistar, after body weight normalisation and with oral exposure, the median lethal dose of queens differed from males but not workers (Table 2; Fig. 2). This suggests that body weight was a key, but not the only factor explaining the differences in sensitivity across castes for this substance.

### Body weight does not explain pesticide sensitivity across sexes in *B. terrestris*

Bumble bee males were more sensitive than workers to sulfoxaflor and Amistar before doses were normalised by body weight (Table 2; Fig. 2). Such differences were still significant after doses were normalised by fresh weight. This suggests that bumble bee males were less resilient than workers to pesticide exposure for reasons other than differences in body weight.

### *O. bicornis* males and females are equally sensitive to sulfoxaflor and Amistar

*Osmia bicornis* females and males were equally sensitive to sulfoxaflor upon oral and contact exposure, both before and after pesticide intake was normalised by body weight (Table 2; Fig. 2), despite females being on average 0.97 times heavier than males (Supplementary material S2 Table S1).

Similarly, upon oral exposure to Amistar, *Osmia bicornis* females and males showed equal sensitivity before and after dose normalisation (Table 2; Fig. 2).

### Azoxystrobin and glyphosate (as active substance or formulation) do not cause mortality in limit tests across castes and sexes of *O. bicornis* and *B. terrestris*

Azoxystrobin and glyphosate were either tested as active substance or formulation using a single, high (i.e., ‘limit’) dosing regime. None of the test items caused significant mortality levels (Table 3). Additional details on the methodology and results of the limit tests are reported in the supplementary material (S6).

**Table 3:** The toxicity of Amistar and glyphosate (as active substance or formulation) across sexes and castes in the limit test designs.

Species	Sex/caste	Test item	Route	Timepoint (h)	NOEL* µg a.s./bee (ng a.s./mg bodyweight)	p-value (Fisher’s exact test)
*B. terrestris* ssp. *terrestris*	Worker	Amistar	Contact	24	200 (836)	1
	Male	Amistar	Contact	48	100 (376)	1
	Queen	Amistar	Contact	48	200 (228)	1
	Worker	Azoxystrobin	Contact	48	100 (369)	0.297
	Male	Azoxystrobin	Contact	48	100 (323)	0.055
	Queen	Azoxystrobin	Contact	48	100 (109)	1
	Worker	Roundup FL	Contact	24	200 (838)	1
	Male	Roundup FL	Contact	48	200 (721)	0.38
	Queen	Roundup FL	Contact	48	200 (229)	1
*B. terrestris* ssp. *audax*	Worker	Glyphosate	Oral	48	200 (NA)	NA***
	Male	Glyphosate	Oral	48	200 (663)	1
	Queen	Glyphosate	Oral	48	200 (282)	1
*O. bicornis*	Female	Roundup PA	Oral	48	100 (1120)	0.592**
	Female	Amistar	Oral	48	100 (1119)	0.741**
	Male	Roundup PA	Oral	48	100 (2131)	0.759**
	Male	Amistar	Oral	48	100 (2146)	1**
	Female	Azoxystrobin	Contact	48	95.7 (NA)	1
	Male	Azoxystrobin	Contact	48	95.7 (NA)	1

*No Observable Effect Level (highest dose causing no significant mortality).

**Benjamini–Hochberg correction.

***Results published in Straw and Brown^50^.

## Discussion

We hypothesised that *B. terrestris* and *O. bicornis* show different levels of acute pesticide sensitivity (i). Here we show that *O. bicornis* was less sensitive than bumble bees to Amistar, but more sensitive to sulfoxaflor, both before and after dose normalisation by weight. Before dose normalisation, *O. bicornis* females were 10.1 times more sensitive orally to sulfoxaflor than bumble bee workers. This result confirms previous evidence^38^, which reported similar toxicity levels (i.e., LD_50_: 0.083 and 0.009 µg/bee for *B. terrestris* and *O. bicornis* respectively) and a ninefold difference in sensitivity between these two species 48 h after oral exposure to sulfoxaflor.

When comparing the toxicity of sulfoxaflor to *O. bicornis* in our study with the LD_50_ reported in the EU assessment^39^ for *A. mellifera*, *O. bicornis* was found to be 11.2 times orally and 7.4 times topically more sensitive than honey bee workers.

Across dose response experiments we showed that castes and sexes of *B. terrestris*, but not *O. bicornis*, responded differently to pesticide exposure, partially confirming our hypothesis (ii). However, the confidence interval around the sensitivity ratios for *O. bicornis* upon oral exposure was relatively wide, compared to *B. terrestris*. This aspect, which may be partly explained by a less precise LD_50_ estimate, may add uncertainty to the conclusions drawn on the comparative analysis for this species.

Bumble bee queens were more resilient than workers and males to both pesticides and exposure routes. However, once doses were normalised by bee weight, the lower sensitivity of queens relative to workers was much less evident for sulfoxaflor and non-significant for Amistar. Bumble bee males always showed higher sensitivity than workers and queens, both before and after doses were normalised by body weight.

Our results confirmed body weight as a meaningful predictor of acute sensitivity across bumble bee workers and queens. However, consistent with our initial hypothesis (iii), body weight alone did not predict the observed differences in sensitivity across bumble bee castes. A possible explanation for this pattern is that, as queens are larger than workers and males^40^ and are likely to have a thicker cuticle, the diffusion of pesticides through their exoskeleton to the target site may be affected by their robust morphology. Consistent with this hypothesis, toxicokinetic (i.e., the efficiency of cuticular penetration) was shown to regulate neonicotinoid sensitivity in honey bees^41^. Nonetheless, as differences in sensitivity were observed across both oral and contact exposure, we do not expect cuticular penetration to be a meaningful predictor of pesticide toxicity across all exposure routes.

Conversely, we found no evidence that the sensitivity to our model substances varies significantly between sexes of *O. bicornis*, both before and after dose normalisation, despite their differences in body weight (Supplementary material S2).

This evidence confirms that species may differ in patterns of inter-sex sensitivity and builds on previous studies showing that males of *A. mellifera*^29^ and *B. terrestris*^26^, but not *O. bicornis*^42^, were more sensitive than females to neonicotinoid exposure. From a mechanistic standpoint, our results highlight the presence of species- and substance-specific patterns of pesticide sensitivity across sexes and castes, which are not explained by body size alone*.*

Pesticide metabolization may also explain the differences in pesticide sensitivity observed in our experiments. Recent studies characterising the role of cytochrome P450 enzymes in neonicotinoid detoxification attributed species- and substance-specific differences in neonicotinoid sensitivity to their relative presence and site interaction in bee^43–47^. The molecular mechanism behind neonicotinoid detoxification in *O. bicornis* was determined to be underpinned by metabolic activity regulated by cytochrome P450 enzymes within the CYP9BU subfamily^45^, similar, but not identical, to the P450 of the CYP9Q subfamily responsible for this mechanism in bumble bees^44–46^. Thus, the differences in sensitivity between species may be influenced by differential detoxification mechanisms. We are not aware of published studies comparing the expression of cytochrome P450 enzymes across bee sexes and castes. However, we suggest that differences in their relative expression, if any, may predict responses of sexes and castes to pesticide exposure. Consistent with this assumption, bumble bee queens have significantly higher fat body reserves than other castes^48^. As fat bodies express P450 enzymes in bumble bee queens^49^, we hypothesise that the higher fat reserves in queens may parially explain why they were the most resilient caste to pesticide exposure in our study.

As mortality caused by Amistar has previously been connected to gut damage caused by etoxylated alcohols^50^. As we found significant differences across and within species in the sensitivity to Amistar, we show that differential physiological responses may regulate pesticide sensitivity not only to active ingredients but also to co-formulants.

Consistent with previous evidence^35^, we found sulfoxaflor to be more toxic via oral than contact exposure. With oral and contact LD_50s_ of 0.126 and 6.323 µg/bee respectively, we found *B. terrestris* workers to be similarly or less sensitive than honey bee workers to sulfoxaflor^39^. Additionally, *B. terrestris* was 7 times less sensitive than *Bombus impatiens* (LD_50_ = 0.177 µg/bee)^51^ upon oral exposure to sulfoxaflor. This difference may be explained by the lower body weight of *Bombus impatiens* relative to our tested species. The oral toxicity of sulfoxaflor in *B. terrestris* workers fell between the values reported for the same species in a recent regulatory report^39^ for two sulfoxaflor-based formulations (LD_50_ = 0.027 and 0.15 µg/bee for GF-2032 and GF-2626 respectively). Moreover, the contact toxicity estimated in our study was comparable to that reported in the same regulatory document for GF-2032 (LD_50_ = 7.55 µg/bee) but not GF-2626 (LD_50_ = 23.3 µg/bee). While we cannot explain such differences, we hypothesise that the presence of co-formulants in these products may have influenced the toxicity of sulfoxaflor^50^.

*Bombus terrestris* queens and *O. bicornis* females may be directly exposed to pesticides during their life cycles due to their life history traits. *B. terrestris* queens may experience prolonged contact or inhalation exposure while overwintering or nesting in soil^25^. Moreover, reproductive of both species may be orally or topically exposed while foraging for pollen and nectar in early spring^23,25^. This is particularly concerning, since exposure of reproductives may have downstream effects on wild bee population dynamics^52^. Our data provide evidence that the consequently high risk of bumble bee queens to pesticide exposure might be partially buffered by their lower sensitivity upon acute exposure. However, this may not be the case for *O. bicornis*, where females and males showed similar sensitivity to our model substances. Additionally, we found bumble bee males to be significantly more sensitive than workers and queens. As pesticides may impact the mating success and fertility of bumble bee males^53^, their exposure might have negative impacts on population dynamics.

None of the study groups showed an acute mortality response to topical azoxystrobin exposure (Supplementary materials S2 and S6). This corroborates previous findings^50^, in which Amistar’s oral toxicity was found to be due to a co-formulant in the commercial preparation, at higher doses than in the limit tests of our study (supplementary material S6). We also confirm glyphosate (as a.s. or formulation) to have low toxicity for *B. terrestris* and, for the first time, for *O. bicornis*, as no increase in mortality was observed in any caste or sex at doses ranging from 100 to 200 µg/bee (Supplementary materials S2 and S6), exceeding realistic exposure estimates in the field^54^.

Additionally, to our knowledge this is the first attempt to adapt regulatory-ready designs to the testing of bee castes other than workers, which we expect may significantly improve the standardisation of future toxicological studies with queen and male bees.

In conclusion, this is the first systematic characterisation of lethal acute hazards across castes and sexes of two bee species. As such, we believe our data provide an important baseline for further exploration of the mechanisms behind intra-specific differences in sensitivity to pesticides.

## Materials and methods

### Model substances

We used the sulfoximine insecticide sulfoxaflor, the methoxy-acrylate fungicide Amistar (azoxystrobin 250 g/l, Suspension Concentrate, see supplementary methods, S1) and the glycine herbicide glyphosate (as active substance, RoundUp ProActive or RoundUp FL, see supplementary methods, S1) as model substances. Our choice was justified by their widespread use, regulatory status and systemic uptake in plants. Because of these characteristics, the likelihood of bees being exposed in the field was considered similarly plausible across model substances. Additionally, we are not aware of published evidence of the acute toxicity of these substances across castes and sexes of *B. terrestris* and *O. bicornis.*

Sulfoxaflor is a relatively novel insecticide^55–57^, developed to replace or complement the use of older chemical classes against which insect pest populations had developed resistance^57^. However, because of its risks to bees^58^, its uses have been recently restricted in the EU to indoor growing conditions. As a nicotinic acetylcholine receptor (nAChR) competitive modulator, sulfoxaflor targets the same neural receptor as the bee-harming neonicotinoid insecticides^55–57^. Despite evidence that sulfoxaflor may target the nAChR in a distinct way compared to recently banned neonicotinoids^55–57^, these substances were shown to be similarly lethal in acute exposure laboratory settings for individuals of *Apis mellifera*, *B. terrestris* and *O. bicornis*^38^. Additionally, sulfoxaflor was shown to reduce reproduction^59–61^ (but not learning^62,63^) in bumble bees under field-realistic laboratory settings. When applied pre-flowering in a semi-field study design, sulfoxaflor impacted colony growth, colony size and foraging in bumble bees^64^ but not honey bees^65^. Azoxystrobin is a broad-spectrum, systemic fungicide, which has been widely used in agriculture since its first marketing authorisation in 1996^66^*.* Azoxystrobin shows low acute toxicity to honey bees^67^. Azoxystrobin residues were found in nectar and pollen from treated crops^68,69^ and subsequently in the bodies of wild bees^70^. In a semi-field experimental setting, foraging, but not colony growth, was significantly impaired in *B. terrestris* exposed to Amistar (azoxystrobin 250 g/L SC)^64^, while no lethal or sublethal effects could be observed in honey bees^65^ or in *O. bicornis*^71^. However, a recent study showed that, when formulated as Amistar this pesticide induced acute mortality in bumble bees at high doses, which was attributed to the dietary toxicity of the co-formulant C16-18 alcohol ethoxylates^50^.

Glyphosate is a broad-spectrum systemic herbicide and the most widely used pesticide in the world^72^. Products containing glyphosate may be applied to flowering weeds^73^ and contaminate their pollen and nectar^54^, thus driving bee contact and oral exposure. Glyphosate showed low lethal hazards in regulatory-ready laboratory^74^ and semi-field designs when dosed as pure active substance or as MON 52276 (SL formulation containing 360 g glyphosate/L)^75^. A recent study found ready-to-use consumer products containing glyphosate to be lethally hazardous to bumble bees^73^. However, this toxicity was attributed to co-formulants, rather than the active substance itself.

We characterised the acute oral and contact toxicity to *B. terrestris and O. bicornis* of sulfoxaflor, azoxystrobin and glyphosate as either pure active substances or formulation (see supplementary material S2 Table S1). Each test was repeated across castes and sexes of these two species. For bumble bees we used workers, males and gynes (i.e., unmated queens), hereby referred to as queens, whereas for *O. bicornis* we used males and females. Bumble bee experiments were designed following OECD protocols^30,31^, while *O. bicornis* was tested following published^76^ and ring-tested protocols^32^, as an OECD protocol for this latter species is not yet available.

We used a dose response design whenever the test item was found to drive significant mortality in the tested species. In all other cases, a limit test design using a single, high pesticide dose was used. Details on the methods and results of the limit tests are reported in the supplementary materials (S2 and S4).

### Pesticide treatments

All dose response tests were performed with pure sulfoxaflor, while azoxystrobin was tested as a plant protection product (Amistar 250 g a.s./l, SC, Syngenta, UK) in all oral tests, as its solubility in water was insufficient (6.7 mg a.s./L, see EFSA, 2010) to achieve the desired concentrations. Amistar contains co-formulants with hazard classification (54 C16-18 alcohols, ethoxylated < 20% w/w; naphthalenesulfonic acid, dimethyl-, polymer with formaldehyde and methylnaphthalenesulfonic acid, sodium salt < 10% w/w and 1,2-benzisothiazol-3(2H)-one < 0.05% w/w) and other unknown compounds. Therefore, to reduce potential bias due to differences in composition, we used the same production batch of Amistar across all dose response experiments. Pesticide treatments were freshly prepared on each day of exposure by means of serial dilutions of concentrated stock solutions. At this stage, stock and treatment solutions were stored at − 20 °C for subsequent chemical analysis. A subset of these solutions was analysed for absolute quantification of active substance (Supplementary material S3). Each dose response included a minimum of 5 serial dilutions of the test item spaced by a factor ≤ 2, in addition to one untreated control, an acetone control (in cases where the test substance was dissolved in such solvent) and a positive control (dimethoate).

Further details on the test solutions and dosing regimen are given in the supplementary materials (S1 and S2).

### *B. terrestris*: oral and contact toxicity tests

Oral exposure experiments were carried out in the UK using *B. terrestris* ssp. *audax*, while contact exposure experiments were conducted in Estonia using *B. terrestris* ssp. *terrestris*. Our design did not specifically investigate how responses to pesticide exposure may vary across these two subspecies.

#### Test organisms and conditions

For oral exposure, *B. terrestris* ssp. *audax* colonies were purchased from a local supplier (Agralan, UK) as queen-right standard hives (i.e., > 80 workers). Upon arrival, bees were screened for the most prevalent gut parasites (*Apicystis bombi*, *Crithidia* spp., and *Nosema* spp.) through microscopic examination of faecal samples (n = 5 per colony box) using a Nikon eclipse (50i) compound microscope at 400X magnification. No infections were detected. Males were either collected from the same commercial colonies described above or, when this was not possible, by direct purchase (Agralan, UK). All queens were collected from queen-right colonies provided *pro bono* by a commercial supplier (Koppert, Slovakia), which were screened using the same methods described above.

For the contact exposure experiments, *B. terrestris* ssp. *terrestris* were purchased as queen‐right standard hives (i.e., > 80 workers; A.M. Ozoli, Latvia), while males and queens of the same subspecies were obtained from queen-less boxes (A.M. Ozoli, Latvia). These bees were not microscopically examined for parasite infections. However, their health status was visually checked upon arrival.

For both exposure routes, bees were kept and tested in complete darkness in a rearing room at 26˚C and the humidity at ∼ 60%. Bee handling was undertaken under red light. Before and after exposure, orally exposed bees were fed ad libitum inverted syrup (45% w/w, Thornes, UK), while topically exposed bees were given ad libitum access to sucrose syrup (50% w/v). Prior to exposure, bees were given a provision of fresh-frozen, honey bee-collected pollen pellets (Agralan, UK and A. M. Ozoli, Latvia for oral and contact tests respectively). We could not analyse all pollen batches used across bumble bee acute oral exposure studies. However, screening of this pollen source found only low levels of miticides used in honeybee hives (results not shown). The pollen used in contact exposure experiments was obtained by a local supplier who distributes pollen from certified organic beekeeping. This pollen source was not analysed. However, considering it was sourced from organically managed apiaries, it is considered unlikely that this pollen source was contaminated with relevant concentrations of our model agrochemicals.

#### Experimental design

For both exposure routes and 1 day prior to their chemical exposure, bees of unknown age were weighed to the nearest milligram, before being individually housed in plastic cages (Nicot, Nicotplast, FR) for acclimatisation. At this stage, exceptionally large or small bees^30,31^ were visually excluded. On the following day, bees were allocated to treatments by colony of origin and body weight. When this was not possible (i.e., directly purchased males), the experimental design only controlled for body weight effects. Depending on the experiment, 3 to 9 colonies per test were used.

For oral exposure, following 4 h starvation, bees were given a 40 µL provision of pesticide-spiked or untreated sucrose syrup through a 2 ml syringe (Becton Dickinson, USA) with the tip removed. Four hours after exposure, syringes were visually inspected to ensure complete consumption of the treatment solution. At this stage, bees that did not consume the entire pesticide provision were removed. Across oral dose–response experiments, the average initial sample size per treatment group (i.e., test item and negative control) was 33 for workers, 34 for males and 43 for queens. Upon exclusion of unexposed bees, the average final sample size was 29 for workers (range: 20–35), 24 for males (range: 15–33) and 20 for queens (range: 7–36) (Supplementary material S2, Table S1). The lower limit of the range of sample sizes reported above corresponded to most concentrated treatment group in the Amistar experiments. For this compound we observed a dose-dependent feeding inhibition, which we attributed to the high dosing and viscosity of the treatment solution.

For contact exposure, following cold anesthetization, a droplet of treatment solution that was either spiked or not with the test solution, was applied to the dorsal side of the thorax (i.e., mesonotum) of each bee. The treatment volume was adjusted by bee size, with workers and males being exposed via a 2 µL droplet while queens were exposed to 4 µL. Upon topical exposure, bees were given ad libitum access to sucrose syrup through a 2 ml syringe with the tip removed (Terumo, Belgium).

Mortality was recorded at 24, 48, 72 and 96 h post-exposure. In dose response contact experiments, we tested 46 workers, 40 males and 30 queens per treatment group except for the untreated and solvent controls in the queen experiment, in which 15 individuals were tested per control group (Supplementary material S2, Table S1). Across experiments we tested 3685 bumblebees (workers: 1397; males: 1283 and queens: 1005; Supplementary material, S2).

### *Osmia bicornis*: oral and contact toxicity tests

All *O. bicornis* experiments were carried out in Germany.

Diapausing *O. bicornis* males and females were purchased from a commercial rearing facility (Pollinature GhmB, Konstanz, DE). Upon arrival, cocoons were visually sorted by sex and stored in darkness at 4 °C in plastic bags.

To induce emergence from diapause, cocoons were placed in an incubator (Memmert, DE) at 21 ± 1 °C and 40% relative humidity. Cocoons were checked twice daily and, upon emergence, bees were transferred back to 4 °C to keep them dormant for maximum 4 days until test initiation.

Emerged males or females were allocated by day of hatching to rearing plastic boxes (27 * 14 * 16 cm) in groups of 10–20. Two feeders (Eppendorf 2 mL tubes with a 2 mm hole at the bottom) with a visual cue in the form of a petal (*Brassica rapa* or *Diplotaxis tenuifolia*) were provided in each cage. After ∼ 4 h of group housing in daylight, the now meconium-free bees were weighed to the nearest milligram and transferred to individual Nicot cages. Unusually small or large individuals were removed upon visual inspection and randomization was performed so that the treatments had and equal distribution of age classes (i.e., days after emergence) and body weights (mg). Bees were then left to starve at 21 °C overnight, after which each bee was presented with 20 µL of sucrose syrup containing pesticide or control treatment. The dose was presented in a cut-off tip of a 0.5 mL Eppendorf tube with a petal identical to the ones used in the hoarding cage in order to stimulate feeding behaviour. Using this method, we visually confirmed consumption, and found 74.5% of males and 88.3% of females (Supplementary material S2 Table S1; mean ratio across treatment groups) consumed the solution within 3 h. Bees that had not consumed the entire droplet within 3 h were considered non-feeders and were excluded. Consequently, across oral dose–response experiments, the average initial sample size per treatment group (i.e., test item and negative control) was 32 for females and 36 for males, while the final sample size was 27 for females [range 33–15], 245 for males [32–13]; Supplementary material S2, Table S1). Similar to bumble bees, the lower limit of the range of sample sizes reported above correspond to Amistar treatments. For this compound we observed feeding inhibition which we attributed to the high dosing and viscosity of the treatment solution.

For contact exposure, the above experimental procedure was repeated up to the allocation to treatments, after which bees were immediately cold-anaesthetized and topically exposed to the treatments. 1µL of solution was pipetted onto the dorsal part of the thorax between the wing-bases and bees were immediately returned to their individual Nicot holding cages. Across dose response contact experiments, a total of 30 individuals were included per treatment group dosed with the test item, and 20 individuals were included per control group (untreated control and solvent control; Supplementary material S2, Table S1). During the test phase, bees were kept in individual Nicot cages of the same design as described above. Bees were kept in an incubator at 21 °C, 16:8 h light:dark cycle for the remainder of the test. 50% w/v sugar solution was provided ad libitum in honey bee queen feeding cups (Nicot, Nicotplast, FR), covered by a metal mesh at the bottom of the cage. Mortality was recorded at 24, 48, 72 and 96 h post-exposure.

Across experiments we tested 1668 *O. bicornis* (females: 819 and males: 849; supplementary material, S2).

### Data curation and statistical analysis

We defined the last observation timepoint at which increases in mortality were lower than 10% as a steady-state mortality level. We used this timepoint as baseline for hazard characterisation and comparative analysis across bee sexes, castes and species. This approach was preferred over the arbitrary selection of a fixed time point (e.g., 48 h) across experiment, as it enabled a more realistic characterisation of acute hazards. This choice was compliant with standard OECD methods^30,31^, which recommend extending the test duration beyond 48 h and up to 96 h whenever the mortality rate in the treated groups increases by ≥ 10% within a 24 h timeframe, whilst control mortality remains at acceptable (low) levels. The rationale behind the OECD recommendation is that the onset of lethal effects upon acute exposure may be delayed in time, in which case, selecting 48 h as timepoint for LD_50_ derivation may underestimate hazard.

However, when *O. bicornis* females were orally exposed to sulfoxaflor, the latest timepoint at which the control mortality was below 15% was 48 h. Therefore, for this latter test the LD_50_ was calculated at 48 h. For consistency, the same timepoint was selected for *O. bicornis* males exposed to sulfoxaflor^38^. Similarly, for Amistar, 48 h was selected as a valid timepoint for both sexes, as the control mortality of *O. bicornis* males exceeded 15% at 72 h.

To minimise statistical bias in comparative analyses, we used the same statistical model for dose response analysis across all experiments. After selection of mortality timepoints, dose responses were fitted using a log-normal model, based on which we estimated the median lethal doses (LD_50s_) and its asymptotic-based *delta* confidence interval*.* Analyses were first carried out by expressing doses as a function of the pesticide intake per bee (i.e., µg/bee) and then by normalising pesticide intake by fresh bee weight (i.e., ng/mg body weight). This enabled us to determine if, and to what extent, differences in bodyweight predicted the responses to pesticide exposure across bee castes, sexes and species. Doses were expressed as measured concentration whenever the chemical analysis of the test solutions deviated from the nominal concentration by more than 20%^77^ (Supplementary material S3). Mortality rates were corrected using Schneider-Orelli’s formula whenever control mortality at the steady-state timepoint exceeded 5%. Whenever acetone was used, we tested a solvent (acetone) control and a water control in parallel^37^, which were compared using a Fisher’s exact test. No difference in mortality between the two groups was observed.

Pesticide hazards across sexes and castes were compared with LD_50_ values (Table 1, Supplementary material S5 Fig. S3), which were used to calculate sensitivity ratios (SR) and relative confidence intervals using the *comped* function in *drc*^33,34^. Statistical analyses and data visualisation were performed in R^78^ using the packages *dplyr*^79^, *drc*^71^ and *ggplot2*^80^.

For the limit tests, mortality rates were compared between controls and exposure groups using a Fisher’s exact test. Whenever appropriate, p-values were adjusted using the Benjamini–Hochberg correction.

## Supplementary Information

Supplementary Information.

## Data Availability

The datasets generated during and analysed during the current study are available in the Zenodo repository, 10.5281/zenodo.7065152.
