# Author Correction: Intra-specific variation in sensitivity of *Bombus terrestris* and *Osmia bicornis* to three pesticides

**DOI:** 10.1038/s41598-022-25623-2

**Published:** 2022-12-06

**Authors:** Alberto Linguadoca, Margret Jürison, Sara Hellström, Edward A. Straw, Peter Šima, Reet Karise, Cecilia Costa, Giorgia Serra, Roberto Colombo, Robert J. Paxton, Marika Mänd, Mark J. F. Brown

**Affiliations:** 1 Centre for Ecology, Evolution & Behaviour, Department of Biological Sciences, School for Life Sciences and the Environment, Royal Holloway University of London, Egham, UK; 2 Pesticide Peer Review Unit, European Food Safety Authority (EFSA), via Carlo Magno 1A, 43126 Parma, Italy; 3 Chair of Plant Health, Institute of Agricultural and Environmental Sciences, Estonian University of Life Sciences, Tartu, Estonia; 4 General Zoology, Institute for Biology, Martin Luther University Halle-Wittenberg, Halle (Saale), Germany; 5 Department of R&D, Koppert s.r.o., Nové Zámky, Slovakia; 6 CREA Research Centre for Agriculture and Environment, via di Corticella 133, 40128 Bologna, Italy

Correction to: *Scientific Reports* 10.1038/s41598-022-22239-4, published online 15 October 2022

The Competing interests section in the original version of this Article was incorrect.

“The authors declare no competing interests.”

now reads:

“The authors declare no competing interests. The views expressed in this article are the authors only and do not reflect the views of the European Food Safety Authority.”

The original Article has been corrected.

