# Supplementary material for: Intra-specific variation in sensitivity of Bombus terrestris and Osmia bicornis to three pesticides
Source: Sci Rep. 2022 Oct 15;12:17311. doi: 10.1038/s41598-022-22239-4 (PMC9569340; doi:10.1038/s41598-022-22239-4)
Supplement: Supplementary file 1 — Supplementary Information. [file 41598_2022_22239_MOESM1_ESM.docx]

**Supplementary material**

**Intra-specific variation in sensitivity of *Bombus terrestris* and *Osmia bicornis* to three pesticides**

Alberto Linguadoca*^1,2^, Margret Jürison*^#3^, Sara Hellström*^4^, Edward A. Straw^1^, Peter Šima^5^, Reet Karise^3^, Cecilia Costa^6^, Giorgia Serra^6^, Roberto Colombo^6^, Robert J. Paxton^4^, Marika Mänd^3^, Mark J. F. Brown^1^

^1^Centre for Ecology, Evolution & Behaviour, Department of Biological Sciences, School for Life Sciences and the Environment, Royal Holloway University of London, Egham, UK.

^2^Pesticide Peer Review Unit. European Food Safety Authority (EFSA), via Carlo Magno 1A, Parma 43126, Italy

^3^Chair of Plant Health, Institute of Agricultural and Environmental Sciences, Estonian University of Life Sciences, Tartu, Estonia.

^4^General Zoology, Institute for Biology, Martin Luther University Halle-Wittenberg, Halle (Saale), Germany.

^5^Department of R&D, Koppert s.r.o., Nové Zámky, Slovakia.

^6^CREA Research Centre for Agriculture and Environment, via di Corticella 133, 40128, Bologna, Italy

*These authors contributed equally: Alberto Linguadoca, Margret Jürison, Sara Hellström.

^#^Corresponding author; email: margret.jyrison@emu.ee

**Table of contents**

[***S1 Supplementary methods. Protocol used for the preparation of the test solutions 3***](#_heading=h.gjdgxs)

[***S2 Supplementary methods. Test design, dose selection and sample size used across dose-response and limit tests 5***](#_heading=h.30j0zll)

[***S3 Supplementary methods. Chemical analyses 1***](#_heading=h.3dy6vkm)***2***

[***S4 Supplementary methods. Limit tests 1***](#_heading=h.1fob9te)***6***

[***S5 Supplementary figures. Dose-response analyses for Sulfoxaflor and Amistar 1***](#_heading=h.3znysh7)***7***

[***S6 Supplementary results. Limit tests 2***](#_heading=h.2et92p0)***0***

[***S7 Bibliographical references 2***](#_heading=h.tyjcwt)***1***

**S1 Supplementary methods. Protocol used for the preparation of the test solutions**

*Test solutions: Bombus terrestris, oral exposure*

Water-based, concentrated stock solutions were prepared using analytical grade sulfoxaflor (CAS n° 946578-00-3, 99.4% purity, Chemservice, USA), dimethoate (CAS Number: 60-51-5, purity ≥ 98.0 %, Pestanal, Sigma Aldrich, UK) and glyphosate (1071-83-6, purity ≥ 98.0 %, Pestanal, Sigma Aldrich, UK).

Additionally, due to the poor solubility of azoxystrobin, a commercial formulation (Amistar 250 g a.i./l, SC, Syngenta, UK, formulation identifiers UK MAPP: 18039, Syngenta ID: A12705B, Agrigem Ltd) was used as a concentrated stock. Each of these solutions was further diluted in 30% w/w syrup to the target concentration. Control solutions were prepared using the same concentration of untreated sucrose syrup. Where relevant (i.e., dose response designs) treatment solutions were prepared by means of serial dilutions in 30% (w/w) sucrose syrup. Dose selection was informed by range-finding, pilot tests (results not shown).

*Test solutions:* Bombus terrestris*, contact exposure*

Acetone-based, concentrated stock solutions were prepared using analytical grade sulfoxaflor (CAS n° 946578-00-3, 99.4% purity, Chemservice, USA) and dimethoate (CAS number: 60-51-5, 99.9% purity, Honeywell Fluka). For azoxystrobin exposure, the commercial formulation Amistar (as above) and analytical grade azoxystrobin (CAS number: 131860-33-8, purity ≥ 98.0 %, Pestanal, Sigma Aldrich, UK) was used as a concentrated stock. Due to the insufficient solubility of glyphosate in water, a commercial formulation Roundup FL (450 g a.i/L, Monsanto Crop Sciences A/S, Denmark) was used as a concentrated stock.

Stock solutions were prepared by dissolving pure sulfoxaflor (10 mg), dimethoate (100 mg) and azoxystrobin (100 mg) in acetone (0.2, 2 and 2 ml respectively). Commercial formulation Amistar and Roundup FL were diluted in water to the desired concentration. For control solutions, acetone and water were used. All pipetting steps were done on ice to minimise evaporation. Where relevant (i.e., dose response designs) treatment solutions were prepared by means of serial dilutions. Dose selection was informed by range-finding, pilot tests (results not shown).

All solutions, including the controls were spiked with a wetting agent (Triton-X-100, 0.1%, Sigma Aldrich) to ensure homogenous distribution of the treatment.

*Test solutions:* Osmia bicornis, *oral and contact exposure*

Acetone-based concentrated stock solutions were prepared using analytical grade sulfoxaflor (Lot n° 9700100, 99.4% purity, Chemservice, USA). 10 mg of sulfoxaflor in powder form was solved in 2 mL of acetone and subsequently serially diluted in the same solvent to the desired test concentration. For oral tests, the desired concentration was added to a 25% v/w sugar solution, creating a test solution with 1% acetone solvent. For topical exposure, the acetone-based solution was used directly. All pipetting steps were done on ice to prevent excess evaporation. For azoxystrobin oral exposure, the commercial formulation Amistar (Amistar 250 g a.i./l, SC, Syngenta, UK) was serially diluted by a factor of two in distilled water to the desired concentration and mixed 1:1 with 50% w/v sugar solution in order to achieve a 20 µL test solution containing 25% w/v sugar. For contact exposure, 2 µL pure azoxystrobin (CAS n° 131860-33-8, purity 98.7%, TraceCERT, Sigma Aldrich, Switzerland) dissolved in acetone was used directly. Glyphosate in its commercial formulation (RoundUp ProActive 480 g a.i/L, Bayer inc., Germany) was used as stock solution for oral and contact exposure in limit tests. RoundUp was mixed with water to the desired concentration and mixed 1:1 with 50% w/v sugar solution, creating a 25% w/v sugar solution with the test substance. For contact exposure, the formulation was diluted with water to the desired concentration, and a wetting agent (Triton-X-100, 0.1%, Sigma Aldrich) was added as a surfactant.

Dimethoate (CAS n° 60-51-5, purity 98%, Pestanal, Sigma Aldrich, USA) solved in acetone was used as a positive control in all trials.

**S2 Supplementary methods. Test design, dose selection and mortality across dose-response and limit tests**

*Dose-response designs*

**Table S1:** Dose-response designs of tested items, doses, numbers of individuals used, individual body weights and standard deviations for bees included in the final sample size, and mortality rates per bee species sex/caste

|  | **Sulfoxaflor LD_50_ *Bombus terrestris* contact** | | | | | | | | |
| --- | --- | --- | --- | --- | --- | --- | --- | --- | --- |
| **Sex/caste** | | **Test item** | **Dose**  **µg a.s./bee (ng a.s./mg bodyweight)** | **Initial sample size** | **Final sample size*** | **Steady-state mortality timepoint** | **Mean body weight (mg [SD])** | **Uncorrected mortality rate** | **Corrected mortality rate** |
| Worker | | Pooled control | 0 (0) | 92 | 92 | 48 | 239 (42) | 0.04 | NA |
|  | | Control (water) | 0 (0) | 46 | 46 | 48 | 239 (42) | 0.02 | NA |
|  | | Acetone | 0 (0) | 46 | 46 | 48 | 239 (42) | 0.07 | NA |
|  | | Sulfoxaflor | 0.1 (0.42) | 46 | 46 | 48 | 236 (42) | 0 | NA |
|  | | Sulfoxaflor | 1 (4.23) | 46 | 46 | 48 | 236 (42) | 0.09 | NA |
|  | | Sulfoxaflor | 5 (21.26) | 46 | 46 | 48 | 235 (44) | 0.48 | NA |
|  | | Sulfoxaflor | 10 (42.17) | 46 | 46 | 48 | 237 (42) | 0.54 | NA |
|  | | Sulfoxaflor | 25 (105.28) | 46 | 46 | 48 | 237 (42) | 0.87 | NA |
|  | | Sulfoxaflor | 50 (210.23) | 46 | 46 | 48 | 238 (42) | 0.96 | NA |
|  | | Dimethoate | 5 (20.83) | 46 | 46 | 48 | 240 (43) | 1 | NA |
| Male | | Pooled control | 0 (0) | 80 | 80 | 72 | 333 (56) | 0 | NA |
|  | | Control (water) | 0 (0) | 40 | 40 | 72 | 336 (57) | 0 | NA |
|  | | Acetone | 0 (0) | 40 | 40 | 72 | 331 (56) | 0 | NA |
|  | | Sulfoxaflor | 0.1 (0.31) | 40 | 40 | 72 | 325 (61) | 0.03 | NA |
|  | | Sulfoxaflor | 1 (3.02) | 40 | 40 | 72 | 331 (58) | 0.73 | NA |
|  | | Sulfoxaflor | 5 (15.03) | 40 | 40 | 72 | 333 (56) | 0.98 | NA |
|  | | Sulfoxaflor | 10 (30.02) | 40 | 40 | 72 | 333 (56) | 1 | NA |
|  | | Sulfoxaflor | 16 (49.2) | 40 | 40 | 72 | 325 (62) | 1 | NA |
|  | | Sulfoxaflor | 25 (74.87) | 40 | 40 | 72 | 334 (57) | 1 | NA |
|  | | Dimethoate | 5 (15.2) | 40 | 40 | 72 | 329 (67) | 1 | NA |
| Queen | | Pooled control | 0 (0) | 30 | 30 | 96 | 789 (73) | 0 | NA |
|  | | Control (water) | 0 (0) | 15 | 15 | 96 | 793 (91) | 0 | NA |
|  | | Acetone | 0 (0) | 15 | 15 | 96 | 786 (52) | 0 | NA |
|  | | Sulfoxaflor | 5 (6.43) | 30 | 30 | 96 | 778 (73) | 0 | NA |
|  | | Sulfoxaflor | 20 (25.65) | 30 | 30 | 96 | 780 (71) | 0.23 | NA |
|  | | Sulfoxaflor | 50 (63.96) | 30 | 30 | 96 | 782 (69) | 0.7 | NA |
|  | | Sulfoxaflor | 100 (127.65) | 30 | 30 | 96 | 783 (70) | 0.8 | NA |
|  | | Sulfoxaflor | 200 (257.66) | 30 | 30 | 96 | 776 (79) | 1 | NA |
|  | | Sulfoxaflor | 400 (507.83) | 30 | 30 | 96 | 788 (69) | 1 | NA |
|  | | Dimethoate | 50 (55.13) | 20 | 20 | 96 | 907 (184) | 1 | NA |
|  | **Sulfoxaflor LD_50_ *Bombus terrestris* oral** | | | | | | | | |
| **Sex/caste** | | **Test item** | **Dose**  **µg a.s./bee (ng a.s./mg bodyweight)** | **Initial sample size** | **Final sample size*** | **Steady-state mortality timepoint** | **Mean body weight (mg [SD])** | **Uncorrected mortality rate** | **Corrected mortality rate** |
| Worker | | Control | 0 (0) | 35 | 33 | 48 | 223 (40) | 0 | NA |
|  | | Sulfoxaflor | 0.05 (0.254) | 35 | 31 | 48 | 205 (35) | 0 | NA |
|  | | Sulfoxaflor | 0.06 (0.29) | 35 | 29 | 48 | 211 (30) | 0 | NA |
|  | | Sulfoxaflor | 0.1 (0.529) | 35 | 33 | 48 | 189 (13) | 0.33 | NA |
|  | | Sulfoxaflor | 0.11 (0.516) | 35 | 35 | 48 | 210 (30) | 0.06 | NA |
|  | | Sulfoxaflor | 0.13 (0.587) | 35 | 34 | 48 | 214 (32) | 0.5 | NA |
|  | | Sulfoxaflor | 0.15 (0.668) | 35 | 31 | 48 | 218 (32) | 0.74 | NA |
|  | | Sulfoxaflor | 0.17 (0.802) | 35 | 32 | 48 | 214 (29) | 0.94 | NA |
|  | | Sulfoxaflor | 0.19 (0.885) | 35 | 30 | 48 | 219 (36) | 0.93 | NA |
|  | | Dimethoate | 4 (16,36) | 35 | 20 | 48 | 245 (25) | 100 | NA |
| Male | | Control | 0 (0) | 30 | 24 | 48 | 241 (48) | 0 | NA |
|  | | Sulfoxaflor | 0.02 (0.094) | 31 | 25 | 48 | 214 (52) | 0.04 | NA |
|  | | Sulfoxaflor | 0.06 (0.251) | 32 | 22 | 48 | 239 (51) | 0.23 | NA |
|  | | Sulfoxaflor | 0.1 (0.457) | 30 | 22 | 48 | 219 (48) | 0.64 | NA |
|  | | Sulfoxaflor | 0.14 (0.602) | 30 | 24 | 48 | 232 (50) | 0.79 | NA |
|  | | Sulfoxaflor | 0.18 (0.77) | 30 | 24 | 48 | 234 (51) | 0.92 | NA |
|  | | Sulfoxaflor | 0.22 (0.906) | 31 | 24 | 48 | 243 (49) | 1 | NA |
|  | | Dimethoate | 4 (15.71) | 30 | 9 | 48 | 255 (51) | 100 | NA |
| Queen | | Control | 0 (0) | 40 | 21 | 96 | 601 (75) | 0 | NA |
|  | | Sulfoxaflor | 0.1 (0.154) | 40 | 19 | 96 | 637 (68) | 0 | NA |
|  | | Sulfoxaflor | 0.2 (0.335) | 40 | 20 | 96 | 603 (97) | 0.05 | NA |
|  | | Sulfoxaflor | 0.44 (0.73) | 40 | 23 | 96 | 601 (77) | 0.48 | NA |
|  | | Sulfoxaflor | 0.86 (1.277) | 40 | 20 | 96 | 673 (221) | 0.9 | NA |
|  | | Sulfoxaflor | 1.51 (2.518) | 40 | 14 | 96 | 601 (61) | 1 | NA |
|  | | Dimethoate | 4 (6.706) | 40 | 24 | 96 | 597 (76) | 0.96 | NA |
|  | **Amistar LD_50_ *Bombus terrestris* oral** | | | | | | | | |
| **Sex/caste** | |  | **Dose**  **µg a.s./bee (ng a.s./mg bodyweight)** | **Initial sample size** | **Final sample size*** | **Steady-state mortality timepoint** | **Mean body weight (mg [SD])** | **Uncorrected mortality rate** | **Corrected mortality rate** |
| Worker | | Control | 0 (0) | 30 | 29 | 96 | 237 (48) | 0 | NA |
|  | | Amistar | 40 (175) | 30 | 28 | 96 | 229 (50) | 0 | NA |
|  | | Amistar | 80 (339) | 30 | 29 | 96 | 236 (46) | 0.1 | NA |
|  | | Amistar | 160 (671) | 30 | 24 | 96 | 239 (50) | 0.17 | NA |
|  | | Amistar | 320 (1303) | 30 | 26 | 96 | 246 (48) | 0.58 | NA |
|  | | Amistar | 640 (2620) | 30 | 24 | 96 | 244 (53) | 0.96 | NA |
|  | | Amistar | 1280 (5261) | 30 | 20 | 96 | 243 (54) | 1 | NA |
|  | | Dimethoate | 4 (16.77) | 30 | 29 | 96 | 239 (49) | 1 | NA |
| Male | | Control | 0 (0) | 37 | 33 | 72 | 288 (57) | 0 | NA |
|  | | Amistar | 40 (144) | 37 | 28 | 72 | 278 (63) | 0 | NA |
|  | | Amistar | 80 (290) | 37 | 26 | 72 | 276 (54) | 0.12 | NA |
|  | | Amistar | 160 (541) | 37 | 25 | 72 | 296 (67) | 0.48 | NA |
|  | | Amistar | 320 (1147) | 37 | 21 | 72 | 279 (50) | 1 | NA |
|  | | Amistar | 640 (2156) | 37 | 18 | 72 | 297 (66) | 0.94 | NA |
|  | | Amistar | 1280 (4524) | 37 | 15 | 72 | 283 (61) | 1 | NA |
|  | | Dimethoate | 4 (13.77) | 37 | 20 | 72 | 291 (55) | 1 | NA |
| Queen | | Control | 0 (0) | 55 | 36 | 96 | 744 (157) | 0 | NA |
|  | | Amistar | 175 (244) | 45 | 25 | 96 | 717 (96) | 0.04 | NA |
|  | | Amistar | 350 (479) | 45 | 19 | 96 | 731 (84) | 0.11 | NA |
|  | | Amistar | 700 (917) | 45 | 18 | 96 | 763 (190) | 0.72 | NA |
|  | | Amistar | 1400 (1874) | 45 | 16 | 96 | 747 (95) | 0.81 | NA |
|  | | Amistar | 2800 (4107) | 45 | 7 | 96 | 682 (91) | 1 | NA |
|  | | Dimethoate | 4 (5.48) | 45 | 28 | 96 | 730 (78) | 0.96 | NA |
|  | **Sulfoxaflor LD_50_ *Osmia bicornis* contact** | | | | | | | | |
| **Sex/caste** | |  | **Dose**  **µg a.s./bee (ng a.s./mg bodyweight)** | **Initial sample size** | **Final sample size*** | **Steady-state mortality timepoint** | **Mean body weight (mg [SD])** | **Uncorrected mortality rate** | **Corrected mortality rate** |
| Female | | Pooled control | 0 (0) | 40 | 40 | 96 | 86 (18) | 0.1 | NA |
|  | | Control (water) | 0 (0) | 20 | 20 | 96 | 85 (19) | 0.15 | NA |
|  | | Acetone | 0 (0) | 20 | 20 | 96 | 86 (18) | 0.05 | NA |
|  | | Sulfoxaflor | 0.25 (2.833) | 30 | 30 | 96 | 88 (17) | 0.87 | 0.85 |
|  | | Sulfoxaflor | 0.125 (1.409) | 30 | 30 | 96 | 89 (17) | 0.77 | 0.74 |
|  | | Sulfoxaflor | 0.063 (0.699) | 30 | 30 | 96 | 89 (17) | 0.5 | 0.44 |
|  | | Sulfoxaflor | 0.031 (0.349) | 30 | 30 | 96 | 90 (18) | 0.43 | 0.37 |
|  | | Sulfoxaflor | 0.016 (0.173) | 30 | 30 | 96 | 90 (18) | 0.37 | 0.3 |
|  | | Dimethoate | 1(11) | 10 | 10 | 96 | 87 (18) | 1 | NA |
| Male | | Pooled control | 0 (0) | 50 | 49 | 96 | 44 (10) | 0.1 | NA |
|  | | Control (water) | 0 (0) | 30 | 29 | 96 | 43 (10) | 0.07 | NA |
|  | | Acetone | 0 (0) | 20 | 20 | 96 | 38 (10) | 0.15 | NA |
|  | | Sulfoxaflor | 0.25 (5.697) | 30 | 27 | 96 | 44 (11) | 0.96 | 0.96 |
|  | | Sulfoxaflor | 0.125 (2.709) | 26 | 26 | 96 | 46 (11) | 0.81 | 0.80 |
|  | | Sulfoxaflor | 0.063 (1.458) | 30 | 30 | 96 | 43 (10) | 0.7 | 0.7 |
|  | | Sulfoxaflor | 0.031 (0.723) | 30 | 29 | 96 | 43 (10) | 0.38 | 0.38 |
|  | | Sulfoxaflor | 0.016 (0.366) | 30 | 29 | 96 | 43 (10) | 0.45 | 0.45 |
|  | | Dimethoate | 1(22) | 15 | 15 | 96 | 43 (10) | 1 | NA |
|  | **Sulfoxaflor LD_50_ *Osmia bicornis* oral** | | | | | | | | |
| **Sex/caste** | |  | **Dose**  **µg a.s./bee (ng a.s./mg bodyweight)** | **Initial sample size** | **Final sample size*** | **Last valid time point** | **Mean body weight (mg [SD])** | **Uncorrected mortality rate** | **Corrected mortality rate** |
| Female | | Pooled control | 0 (0) | 58 | 49 | 48** | 88 (16) | 0.14 | NA |
|  | | Control (water) | 0 (0) | 36 | 28 | 48** | 87 (17) | 0.11 | NA |
|  | | Acetone | 0 (0) | 22 | 21 | 48** | 90 (14) | 0.19 | NA |
|  | | Sulfoxaflor | 0.025 (0.283) | 36 | 32 | 48** | 88 (15) | 0.78 | 0.75 |
|  | | Sulfoxaflor | 0.013 (0.137) | 35 | 33 | 48** | 91 (17) | 0.45 | 0.36 |
|  | | Sulfoxaflor | 0.006 (0.071) | 31 | 31 | 48** | 88 (14) | 0.45 | 0.36 |
|  | | Sulfoxaflor | 0.003 (0.035) | 34 | 31 | 48** | 90 (16) | 0.35 | 0.24 |
|  | | Sulfoxaflor | 0.001 (0.018) | 33 | 29 | 48** | 88 (15) | 0.24 | 0.12 |
|  | | Dimethoate | 1(11.8) | 18 | 14 | 48** | 85 (13) | 1 | NA |
| Male | | Pooled control | 0 (0) | 60 | 43 | 48 | 45 (8) | 0.05 | NA |
|  | | Control (water) | 0 (0) | 41 | 30 | 48 | 45 (10) | 0.03 | NA |
|  | | Acetone | 0 (0) | 19 | 13 | 48 | 45 (7) | 0.08 | NA |
|  | | Sulfoxaflor | 0.025 (0.532) | 40 | 32 | 48 | 47 (11) | 0.87 | NA |
|  | | Sulfoxaflor | 0.013 (0.269) | 40 | 28 | 48 | 46 (11) | 0.43 | NA |
|  | | Sulfoxaflor | 0.006 (0.135) | 39 | 29 | 48 | 46 (9) | 0.37 | NA |
|  | | Sulfoxaflor | 0.003 (0.066) | 41 | 27 | 48 | 48 (10) | 0.11 | NA |
|  | | Sulfoxaflor | 0.001 (0.035) | 41 | 32 | 48 | 45 (9) | 0.12 | NA |
|  | | Dimethoate | 1(22.3) | 21 | 16 | 48 | 45 (10) | 1 | NA |
|  | **Amistar LD_50_ *Osmia bicornis* oral** | | | | | | | | |
| **Sex/caste** | |  | **Dose**  **µg a.s./bee (ng a.s./mg bodyweight)** | **Initial sample size** | **Final sample size*** | **Steady-state mortality timepoint** | **Mean body weight (mg [SD])** | **Uncorrected mortality rate** | **Corrected mortality rate** |
| Female | | Control | 0 (0) | 31 | 27 | 48 | 112 (19) | 0.04 | NA |
|  | | Amistar | 640 (5808) | 33 | 26 | 48 | 110 (15) | 0.58 | NA |
|  | | Amistar | 320 (2858) | 29 | 21 | 48 | 113 (16) | 0.24 | NA |
|  | | Amistar | 160 (1424) | 33 | 15 | 48 | 112 (15) | 0.07 | NA |
|  | | Amistar | 80 (703) | 31 | 25 | 48 | 114 (16) | 0.08 | NA |
|  | | Amistar | 40 (352) | 32 | 25 | 48 | 113 (19) | 0 | NA |
|  | | Dimethoate | 1 (8.6) | 3 | 3 | 48 | 116 (5) | 1 | NA |
| Male | | Control | 0 (0) | 32 | 24 | 48*** | 58 (12) | 0.13 | 0 |
|  | | Amistar | 640 (11643) | 33 | 13 | 48*** | 62 | 0.69 | 0.65 |
|  | | Amistar | 320 (6105) | 33 | 17 | 48*** | 59 (7) | 0.47 | 0.39 |
|  | | Amistar | 160 (3061) | 35 | 23 | 48*** | 59 (10) | 0.3 | 0.20 |
|  | | Amistar | 80 (1481) | 32 | 23 | 48*** | 61 (9) | 0.13 | 0.01 |
|  | | Amistar | 40 (762) | 32 | 26 | 48*** | 59 (9) | 0.04 | 0 |
|  | | Dimethoate | Not included |  |  |  |  |  |  |
|  | *Excluding bees who did not consume the whole pesticide provision and incidental deaths (i.e., due to mis-handling or escape)  ** Due to time points 72 and 96 h being invalid for females, the 48 h timepoint was chosen for both sexes  *** Due to time points 72 and 96 h being invalid for males, the 48 h timepoint was chosen for both sexes | | | | | | | | |

*Limit design*

|  | **Contact toxicity of Amistar - limit test with *Bombus terrestris*** | | | | | | | | |
| --- | --- | --- | --- | --- | --- | --- | --- | --- | --- |
| **Sex/caste** | | **Test item** | **Dose**  **µg a.s./bee (ng a.s./mg bodyweight)** | **Initial sample size** | **Final sample size*** | **Steady-state mortality timepoint** | **Mean body weight ( mg [SD])** | **Uncorrected mortality rate** | **Corrected mortality rate** |
| Worker | | Amistar | 200 (836) | 45 | 45 | 24** | 239 (26) | 0 | NA |
|  | | Control | 0 (0) | 45 | 45 | 24** | 240 (27) | 0 | NA |
|  | | Dimethoate | 5 (21) | 45 | 45 | 24** | 238 (26) | 1.00 | NA |
| Male | | Amistar | 100 (376) | 51 | 51 | 48 | 266 (59) | 0.02 | NA |
|  | | Control | 0 (0) | 30 | 30 | 48 | 283 (54) | 0.03 | NA |
|  | | Dimethoate | 5 (17) | 30 | 30 | 48 | 293 (66) | 1.00 | NA |
| Queen | | Amistar | 200 (228) | 20 | 20 | 48 | 877 (174) | 0.00 | NA |
|  | | Control | 0 (0) | 20 | 20 | 48 | 902 (177) | 0.00 | NA |
|  | | Dimethoate | 50 (57) | 20 | 20 | 48 | 872 (170) | 0.95 | NA |
|  | **Contact toxicity of azoxystrobin - limit test with *Bombus terrestris*** | | | | | | | | |
| **Sex/caste** | | **Test item** | **Dose**  **µg a.s./bee (ng a.s./mg bodyweight)** | **Initial sample size** | **Final sample size*** | **Steady-state mortality timepoint** | **Mean body weight ( mg [SD])** | **Uncorrected mortality rate** | **Corrected mortality rate** |
| Worker | | Azoxystrobin | 100 (369) | 50 | 50 | 48 | 271 (44) | 0 | NA |
|  | | Pooled con. | 0 (0) | 92 | 92 | 48 | 239 (42) | 0.04 | NA |
|  | | Acetone | 0 (0) | 46 | 46 | 48 | 239 (42) | 0.02 | NA |
|  | | Control | 0 (0) | 46 | 46 | 48 | 239 (42) | 0.07 | NA |
|  | | Dimethoate | 5 (21) | 46 | 46 | 48 | 240 (43) | 1 | NA |
| Male | | Azoxystrobin | 100 (323) | 50 | 50 | 48 | 309 (72) | 0.06 | NA |
|  | | Pooled con. | 0 (0) | 80 | 80 | 48 | 333 (56) | 0 | NA |
|  | | Acetone | 0 (0) | 40 | 40 | 48 | 336 (57) | 0 | NA |
|  | | Control | 0 (0) | 40 | 40 | 48 | 331 (56) | 0 | NA |
|  | | Dimethoate | 5 (15) | 40 | 40 | 48 | 329 (67) | 1 | NA |
| Queen | | Azoxystrobin | 100 (109) | 30 | 30 | 48 | 915 (121) | 0 | NA |
|  | | Pooled con. | 0 (0) | 30 | 30 | 48 | 790 (73) | 0 | NA |
|  | | Acetone | 0 (0) | 15 | 15 | 48 | 793 (91) | 0 | NA |
|  | | Control | 0 (0) | 15 | 15 | 48 | 786 (52) | 0 | NA |
|  | | Dimethoate | 50 (55) | 20 | 20 | 48 | 907 (184) | 0.95 | NA |
|  | **Contact toxicity of Roundup FL (450 g/l glyphosate, soluble concentrate) - limit test with *Bombus terrestris*** | | | | | | | | |
| **Sex/caste** | | **Test item** | **Dose**  **µg a.s./bee (ng a.s./mg bodyweight)** | **Initial sample size** | **Final sample size*** | **Steady-state mortality timepoint** | **Mean body weight (mg [SD])** | **Uncorrected mortality rate** | **Corrected mortality rate** |
| Worker | | Roundup FL | 200 (838) | 45 | 45 | 24** | 239 (26) | 0 | NA |
|  | | Control | 0 (0) | 45 | 45 | 24** | 240 (28) | 0 | NA |
|  | | Dimethoate | 5 (21) | 45 | 45 | 24** | 238 (26) | 1 | NA |
| Male | | Roundup FL | 200 (721) | 49 | 49 | 48 | 278 (61) | 0.00 | NA |
|  | | Control | 0 (0) | 30 | 30 | 48 | 283 (54) | 0.03 | NA |
|  | | Dimethoate | 5 (17) | 30 | 30 | 48 | 293 (66) | 1.00 | NA |
| Queen | | Roundup FL | 200 (229) | 20 | 20 | 48 | 873 (174) | 0.00 | NA |
|  | | Control | 0 (0) | 20 | 20 | 48 | 902 (177) | 0.00 | NA |
|  | | Dimethoate | 50 (57) | 20 | 20 | 48 | 872 (170) | 0.95 | NA |
|  | **Oral toxicity of glyphosate - limit test with *Bombus terrestris*** | | | | | | | | |
| **Sex/caste** | | **Test item** | **Dose**  **µg a.s./bee (ng a.s./mg bodyweight)** | **Initial sample size** | **Final sample size*** | **Steady-state mortality timepoint** | **Mean body weight ( mg [SD])** | **Uncorrected mortality rate** | **Corrected mortality rate** |
| Worker | |  | Results published in Straw and Brown (2021) | | | | | | |
| Male | | Control | 0 (0) | 37 | 33 | 48 | 288 (57) | 0 | NA |
|  | | Dimethoate | 4 (14) | 37 | 20 | 48 | 291 (55) | 1 | NA |
|  | | Glyphosate | 200 (663) | 60 | 33 | 48 | 302 (60) | 0.03 | NA |
| Queen | | Control | 0 | 55 | 36 | 48 | 744 (157) | 0 | NA |
|  | | Dimethoate | 4 (6) | 45 | 28 | 48 | 730 (78) | 0.96 | NA |
|  | | Glyphosate | 200 (282) | 55 | 18 | 48 | 710 (87) | 0 | NA |
|  | **Oral toxicity of RoundUp ProActive (480 g/L glyphosate) and Amistar - limit test with *Osmia bicornis*** | | | | | | | | |
| **Sex/caste** | | **Test item** | **Dose**  **µg a.s./bee (ng a.s./mg bodyweight)** | **Initial sample size** | **Final sample size*** | **Steady-state mortality timepoint** | **Mean body weight (mg [SD])** | **Uncorrected mortality rate** | **Corrected mortality rate** |
| Female | | Control | 0 (0) | 57 | 48 | 48 | 94 (21) | 0.09 | NA |
|  | | Roundup PA | 100 (1120) | 46 | 37 | 48 | 89 (16) | 0.03 | 0.00 |
|  | | Amistar | 100 (1119) | 58 | 41 | 48 | 89 (17) | 0.12 | 0.03 |
|  | | Dimethoate | 1 (11) | 19 | 15 | 48 | 91 (21) | 1 | 0.91 |
| Male | | Control | 0 (0) | 37 | 32 | 48 | 48 (10) | 0 | 0 |
|  | | Roundup PA | 100 (2131) | 52 | 44 | 48 | 47 (11) | 0.05 | 0.05 |
|  | | Amistar | 100 (2146) | 48 | 37 | 48 | 47 (10) | 0 | 0 |
|  | | Dimethoate | 1 (17) | 20 | 16 | 48 | 59 (8) | 1 | 1 |
|  | **Contact toxicity of azoxystrobin - limit test with *Osmia bicornis*** | | | | | | | | |
| **Sex/caste** | | **Test item** | **Dose**  **µg a.s./bee (ng a.s./mg bodyweight)** | **Initial sample size** | **Final sample size*** | **Steady-state mortality timepoint** | **Mean body weight (mg)** | **Uncorrected mortality rate** | **Corrected mortality rate** |
| Female | | Pooled con. | 0 (0) | 25 | 25 | 48 | NA | 0.08 | NA |
|  | | Control | 0 (0) | 15 | 15 | 48 | NA | 0.07 | NA |
|  | | Acetone | 0 (0) | 10 | 10 | 48 | NA | 0.1 | NA |
|  | | Azoxystrobin | 95.7 (NA) | 30 | 30 | 48 | NA | 0.07 | 0 |
|  | | Dimethoate | 4 (NA) | 10 | 10 | 48 | NA | 1 | 0.92 |
| Male | | Pooled con. | 0 (0) | 30 | 30 | 48 | NA | 0.13 | NA |
|  | | Control | 0 (0) | 15 | 15 | 48 | NA | 0.07 | NA |
|  | | Acetone | 0 (0) | 15 | 15 | 48 | NA | 0.2 | NA |
|  | | Azoxystrobin | 95.7 (NA) | 30 | 30 | 48 | NA | 0.13 | 0.05 |
|  | | Dimethoate | 2 (NA) | 15 | 15 | 48 | NA | 1 | 0.92 |
|  | *Excluding bees who did not consume the whole pesticide provision and incidental deaths (i.e., due to mis-handling or escape)  ** results shown at 24h, as the mortality in the control at 48h exceeded 10% | | | | | | | | |

**S3 Chemical analyses**

*Methods:*

Upon delivery of the treatments on dry ice, solutions were analysed using Ultra-Performance Liquid Chromatography (UPLC) - electrospray ionization tandem mass spectrometry (MS) (Thermo Fisher).

Briefly, for chemical quantification, sulfoxaflor and azoxystrobin calibration curves were derived using a range of pesticide solutions spanning from 2 to 400 ng/ml a.i. These solutions were made by diluting acetone-based stock solution of sulfoxaflor (99.0% purity, Dr. Ehrenstorfer, GmbH), and azoxystrobin (98.7% purity, Dr. Ehrenstorfer, GmbH) in 4mM Ammonium formate with 1% formic acid in water / 4mM Ammonium formate with 1% formic acid in methanol (90v/10v). Additionally, prior to the analysis, Acetamiprid d-3 (CAS n° 1353869-35-8, 98% purity, Sigma-Aldrich) was added to both calibration standards and treatment solutions at the concentration of 100 ng/ml, as an internal standard.

When necessary, particularly concentrated samples were diluted prior to being analysed. The limits of detection (LOD) and quantification (LOQ) of our analytical methodology were identified at 0.001 and 0.002 mg a.i./kg for sulfoxaflor and 0.0001 and 0.0002 mg a.i./kg for azoxystrobin.

Details on the instrument setup and calibration are presented in Tables S2, S3 and Figures S1 and S2.

**Table S2:** The instrument (ULPC and column - Accella 1250 Pump, Thermo Fisher Scientific) setup. Flow rate: 0.5 mL/min; volume injected: 3 µL; column: Raptor ARC-18 2,7 µm 100 x3 mm (Restek), column temperature: 50°C; sample temperature: 5°C; run time: 15 min. Mobile phase A: 4mM Ammonium formate + 1% formic acid in water, mobile phase B: 4mM Ammonium formate + 1% formic acid in methanol.

| Time (min) | % A | %B |
| --- | --- | --- |
| 0.00 | 99 | 1 |
| 2.00 | 99 | 1 |
| 13.00 | 10 | 90 |
| 13.01 | 99 | 1 |
| 15.00 | 99 | 1 |

**Table S3:** The MS Instrument set up (TSQ Quantum Access Max, Thermo Fisher Scientific)

| Compound | quantifier | qualifier | ce | TL | Polarity |
| --- | --- | --- | --- | --- | --- |
| Sulfoxaflor | 278.10 | 104.20 | 33 | 86 | ESI+ |
|  | 278.10 | 154.10 | 28 | 86 | ESI+ |
| Azoxystrobin | 404.12 | 329.1 | 31 | 92 | ESI+ |
|  | 404.12 | 344.12 | 24 | 92 | ESI+ |
|  | 404.12 | 372.1 | 17 | 92 | ESI+ |
| Acetamiprid-d3 (Internal standard) | 226.10 | 90.20 | 32 | 90 | ESI+ |
|  | 226.10 | 126.00 | 15 | 90 | ESI+ |


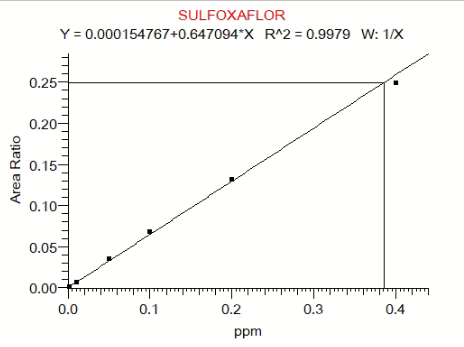


**Figure S1:** Sulfoxaflor calibration curve


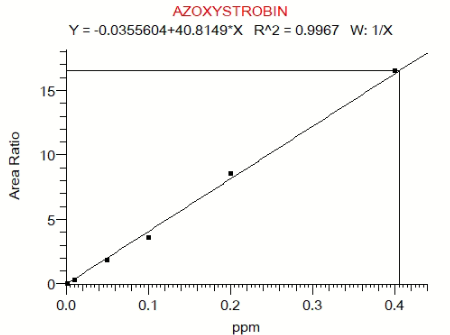


**Figure S2:** Azoxystrobin calibration curve

*Results:*

A number of acetone-based sulfoxaflor solutions evaporated over shipping, hence compromising the chemical quantification (results not shown). Results of the chemical analyses carried out on treatment or stock solutions are presented in Table S4.

**Table S4:** The chemical analyses carried out on a subset of treatment matrixes used in the dose-response experiments

| **Species (exposure)** | **Sex/caste** | **Test item** | **Matrix** | **Method** | **Nominal concentration (mg a.s./L)** | **Measured concentration (mg a.s./L)** |
| --- | --- | --- | --- | --- | --- | --- |
| *B. terrestris* (oral) | male | Sulfoxaflor | Aqueous sucrose syrup | MDP/16 (LC-MS/MS) | 0.5 | 0.485 |
|  |  |  |  |  | 1.5 | 1.421 |
|  |  |  |  |  | 2.5 | 2.33 |
|  |  |  |  |  | 3.5 | 3.432 |
|  |  |  |  |  | 4.5 | 4.103 |
|  |  |  |  |  | 5.5 | 4.922 |
| *B. terrestris* (oral) | queen | Sulfoxaflor | Aqueous sucrose syrup | MDP/16 (LC-MS/MS) | 2.25 | 2.451 |
|  |  |  |  |  | 4.5 | 5.058 |
|  |  |  |  |  | 9 | 10.98 |
|  |  |  |  |  | 18 | 21.471 |
|  |  |  |  |  | 36 | 37.859 |
| *B. terrestris* (oral) | worker | Sulfoxaflor | Aqueous sucrose syrup | MDP/16 (LC-MS/MS) | 1 | 1.3 |
|  |  |  |  |  | 1.5 | 1.53 |
|  |  |  |  |  | 2 | 2.706 |
|  |  |  |  |  | 2.5 | 2.504 |
|  |  |  |  |  | 3 | 3.143 |
|  |  |  |  |  | 3.5 | 3.634 |
|  |  |  |  |  | 4 | 4.299 |
|  |  |  |  |  | 4.5 | 4.843 |
| *B. terrestris* (oral) | male | Amistar | Aqueous sucrose syrup | MDP/16 (LC-MS/MS) | 1000 | 967 |
|  |  |  |  |  | 2000 | 1893 |
|  |  |  |  |  | 4000 | 3929 |
|  |  |  |  |  | 8000 | 7981 |
|  |  |  |  |  | 16000 | 15978 |
|  |  |  |  |  | 32000 | 32820 |
| *B. terrestris* (oral) | queen | Amistar | Aqueous sucrose syrup | MDP/16 (LC-MS/MS) | 4375 | 4718 |
|  |  |  |  |  | 8750 | 7898 |
|  |  |  |  |  | 17500 | 16704 |
|  |  |  |  |  | 35000 | 34146 |
|  |  |  |  |  | 70000 | 68861 |
| *O. bicornis* (all) | all |  |  |  |  |  |
|  |  | Amistar (stock) | water |  | 250000 | 282895 |
|  |  | Azoxystrobin (stock 1:10 dilution) | acetone |  | 500 | 609.88 |

**S4 Supplementary methods. Limit tests**

For chemicals of low toxicity to bees, OECD ^1,2^ recommends using a limit design in place of a full dose-response analysis. Limit tests use a single, high dose of a test chemical, in order to investigate whether median lethal doses of pesticides are above tested levels. When this is confirmed, the tested dose is conservatively used as surrogate LD_50_ in risk assessment. Compared to dose-response designs, limit tests allow to minimise animal testing and investments whenever a full dose-response analysis is not required, or it is not technically feasible (e.g., when desired test concentrations exceed solubility limits).

Based on published evidence ^3–5^ we hypothesised that glyphosate and azoxystrobin are of low toxicity across sexes and castes of *Bombus terrestris* and *Osmia bicornis.* Therefore, we used a limit design to test the acute oral and contact toxicity of glyphosate (as pure active ingredient or formulated as RoundUp ProActive [480 g a.i/L] or Roundup FL [450 g a.i./L SC]) and the contact toxicity of azoxystrobin (as pure active ingredient or formulated as Amistar [azoxystrobin, 250 g/l SC]) across sexes and castes of these two bee species. As explained above, this test design was used whenever a full dose-response could not be characterised because of the low toxicity and poor solubility of the test item. We used a limit test design to rule out large differences in sensitivity across sexes and castes of *B. terrestris* and *O. bicornis*. Limit tests were designed using the same controls as described for the dose-response designs and a single concentration of test item. For *O. bicornis* contact limit tests of azoxystrobin, group housing (5 bees/cage (5*10*9 cm) was used instead of individual housing, and bodyweight was not measured prior to test. Details on the dose preparation, selection and sample sizes are reported under sections S1 and S2 respectively. Limit tests were carried out in compliance with methods described in the main manuscript for dose response designs. However, with a single dose being tested, a dose-response analysis was not possible.

**S5 Supplementary figures. Dose-response analyses for Sulfoxaflor and Amistar**


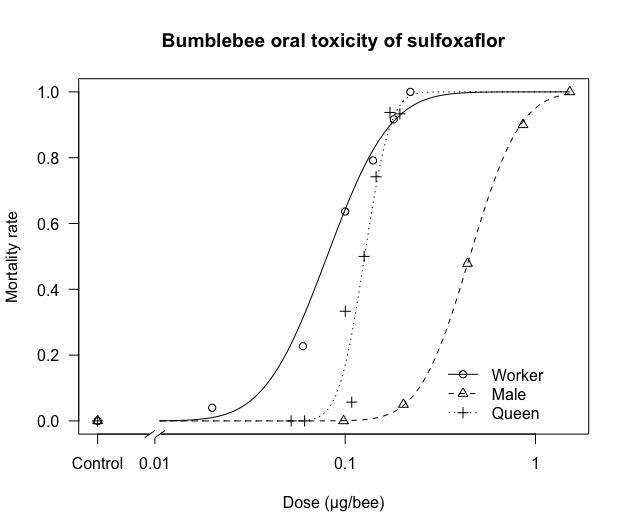

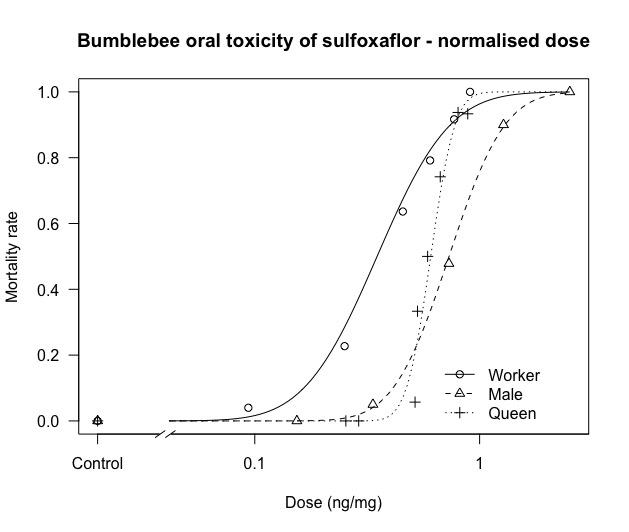

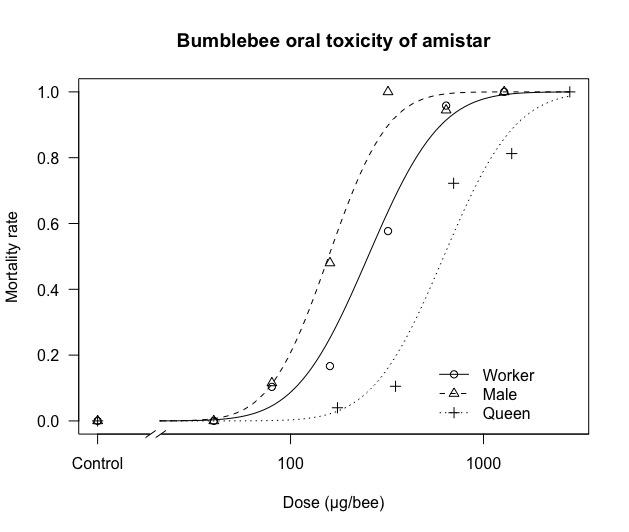

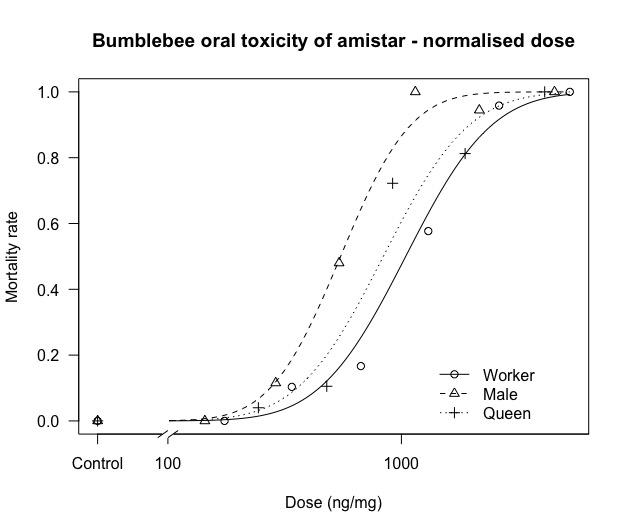

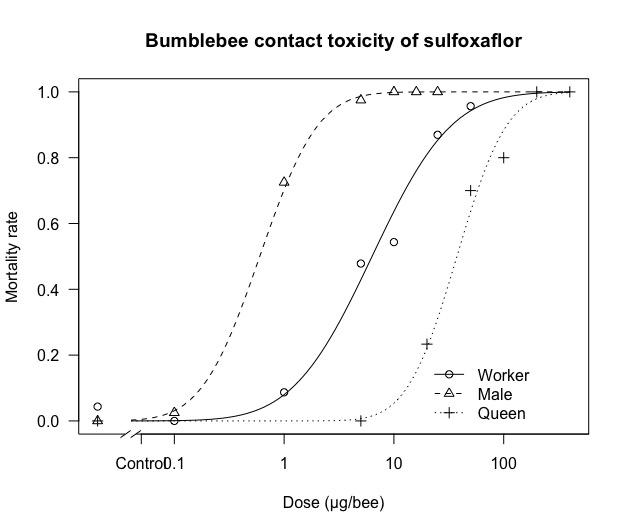

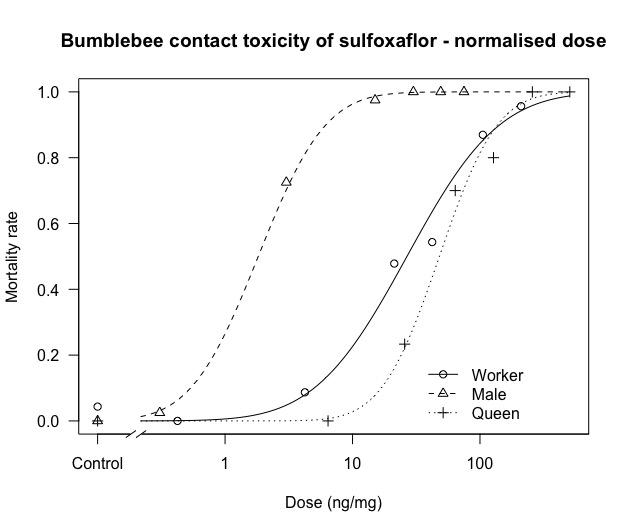

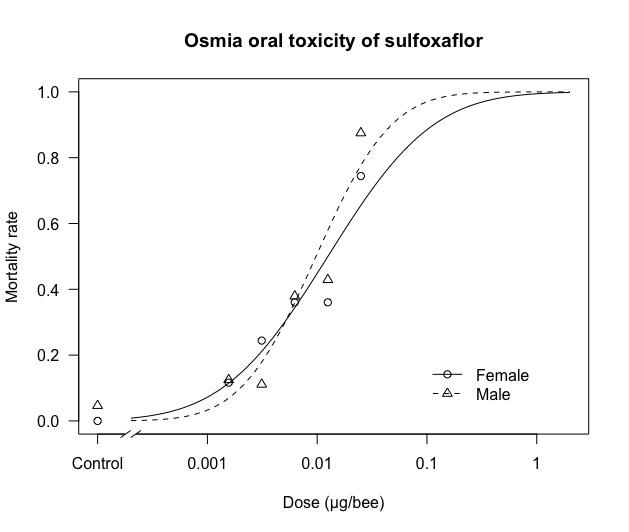

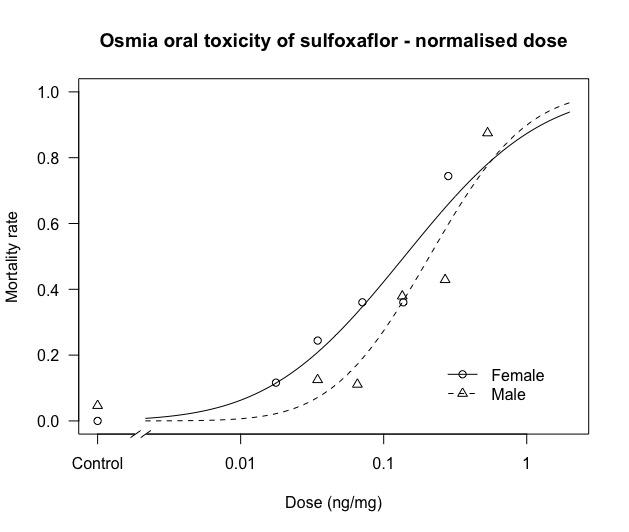

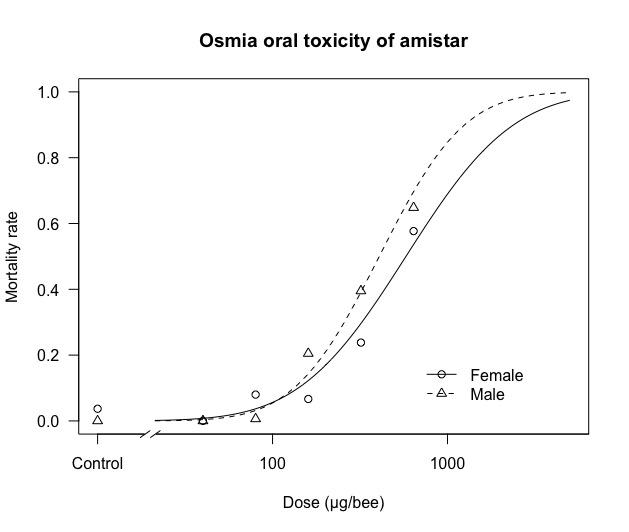

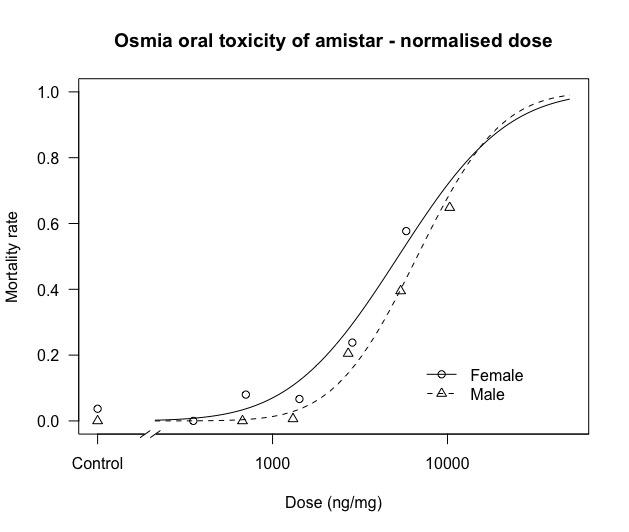


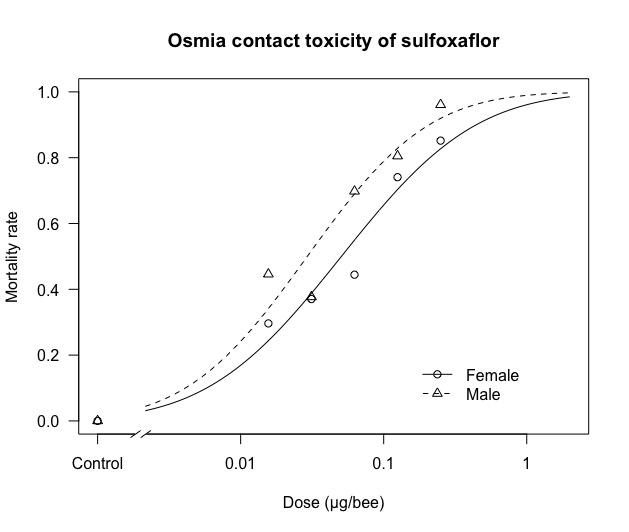

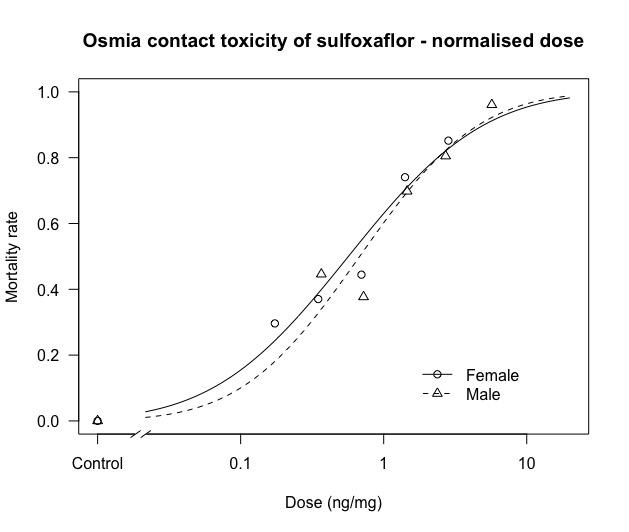


**Figure S3:** Dose-response analyses of oral and contact tests with Sulfoxaflor and Amistar on bumblebee *B. terrestris* and solitary bee *O. bicornis*

**S6 Supplementary results. Limit tests**

No evidence of delayed mortality was observed across experiments. Therefore, the steady state mortality timepoint was considered 48h. At this time point, Dimethoate caused mortality levels above 90% in all limit tests, hence confirming the sensitivity of the test system. Untreated control mortality at 48h (pooled, where relevant) was within acceptable limits in all tests ^1,2,7^, except for two experiments investigating the contact toxicity of Amistar and Roundup FL in bumble bee workers. For these experiments, the control mortality at 24h mortality was well below acceptability thresholds ^2^. Therefore, the mortality level at this time point was reported under section S2.

A detailed summary of the survival of *O. bicornis* and *B. terrestris* exposed to azoxystrobin (formulated or not as Amistar) and glyphosate (formulated or not as Roundup FL or Roundup PA) is reported under section S2. In none of the cases the tested pesticides caused mortality levels allowing to derive an LD_50_.

**S7 Bibliographical references**

1. OECD. *Guideline for the testing of chemicals 247. Bumblebee, acute oral toxicity test.* (2017).

2. OECD. *Guideline for the testing of chemicals 246. Bumblebee, acute contact toxicity test.* (2017).

3. Conclusion on the peer review of the pesticide risk assessment of the active substance azoxystrobin. *EFSA J.* **8**, (2010).

4. EFSA. Conclusion on the peer review of the pesticide risk assessment of the active substance glyphosate. *EFSA J.* **13**, (2015).

5. Straw, E. A. & Brown, M. J. F. Co-formulant in a commercial fungicide product causes lethal and sub-lethal effects in bumble bees. *Sci. Rep.* **11**, 1–10 (2021).

6. R Core Team. R: A language and environment for statistical computing. R Foundation for Statistical Computing, Vienna, Austria. URL https://www.r-project.org/. (2019).

7. EPPO. Environmental risk assessment scheme for plant protection products. Chapter 10: Honey bees. *EPPO Bull.* **40**, 323–331 (2010).
